# Efficacy-shaping nanomedicine by loading Calcium Peroxide into Tumor Microenvironment-responsive Nanoparticles for the Antitumor Therapy of Prostate Cancer

**DOI:** 10.7150/thno.43631

**Published:** 2020-08-02

**Authors:** Di Wu, Zi-Qiang Zhu, Hai-Xiao Tang, Zhi-En Shi, Jian Kang, Qiang Liu, Jun Qi

**Affiliations:** 1 Department of Urology, Xinhua Hospital Affiliated to Shanghai Jiao Tong University School of Medicine, Shanghai 20092, China.; 2 Department of Urology, Huadong Hospital Affiliated to Fudan University, Shanghai 20040, China.

**Keywords:** calcium peroxide, hollow mesoporous silica nanoparticles, prostate cancer, reactive oxygen species, tumor microenvironment

## Abstract

**Rationale:** Prostate cancer has become one of the most threatening malignant tumors in men, leading to an imperative need to develop effective and safe therapies. Because of the unique metabolism of tumor cells, the tumor microenvironment (TME) exhibits distinctive properties compared with normal tissues, among which the pH difference has been utilized as an ideal antitumor strategy. Herein, we introduce a reactive oxygen species (ROS)-controlled-release nanosystem with TME-responsiveness by applying hollow mesoporous silica nanoparticles (HMSNs) as carriers loaded with calcium peroxide (CaO_2_) and coated with polyacrylic acid (PAA) to construct the functional material CaO_2_@HMSNs-PAA. The differences in pH values and exogenous ROS scavenging abilities between the tumor tissue and normal tissues and the dual pH-responsiveness from CaO_2_ and PAA lay a scientific foundation for the application of CaO_2_@HMSNs-PAA in the tumor-selective therapy for prostate cancer.

**Methods:** The morphology and the structure of the nanosystem were characterized by the transmission electron microscope, scanning electron microscope, energy-dispersive X-ray spectroscopy, Fourier transform infrared spectroscopy, zeta potential, dynamic light scattering measurement, low-angle X-ray diffraction patterns and nitrogen adsorption/desorption isotherm. The CaO_2_ loading capacity and release profiles in different buffer solutions were determined by inductively coupled plasma-mass spectrometry. The* in vitro* intracellular uptake of CaO_2_@HMSNs-PAA was explored on the PC-3 prostate cancer cell line *via* confocal laser scanning microscopy. The CCK-8 cell proliferation assay was conducted to evaluate the cytotoxicity of CaO_2_@HMSNs-PAA against PC-3 cells. ROS produced by CaO_2_@HMSNs-PAA was observed by a fluorescence microscope. The flow cytometry was utilized to analyze the apoptosis of PC-3 cells induced by CaO_2_@HMSNs-PAA. The Western blot analysis was performed to detect expressions of critical mitochondria-mediated apoptosis markers in PC-3 cells after incubation with CaO_2_@HMSNs-PAA. The *in vivo* biosafety and antitumor efficacy were evaluated out on BALB/c mice and BALB/c nude mice subcutaneously transplanted with PC-3 cells, respectively.

**Results:** Comprehensive characterizations indicated the successful synthesis of CaO_2_@HMSNs-PAA with significant TME-responsiveness. The experimental results demonstrated that the well-developed nanocarrier could efficiently deliver CaO_2_ to the tumor site and release ROS in response to the decreased pH value of TME, exerting ideal antitumor effects both *in vitro* and *in vivo* by activating the mitochondria-mediated apoptosis pathway. Simultaneously, this nanoplatform caused no detectable damage to normal tissues.

**Conclusions:** After loading into the above nanocomposite, the free CaO_2_ without a significant antitumor effect can exert excellent antitumor efficacy by responsively releasing ROS under the acidic TME to induce the mitochondria-mediated apoptosis *via* remarkable oxidative stress and simultaneously minimize damages to normal tissues. The current study presents a new concept of “efficacy-shaping nanomedicine” for the tumor-selective treatment of prostate cancer.

## Introduction

Prostate cancer (PCa) has been one of the most threatening malignant tumors in men 1, so it is of great significance to develop effective therapeutics against this disease. Currently, for the treatment of prostate cancer and especially the advanced prostate cancer, conventional radiotherapy and chemotherapy are still important treatments. Because of the difficulties in effectively distinguishing the tumor tissue from normal tissues during treatment implementation and a variety of serious side damages to normal tissues, the comprehensive efficacy is unsatisfactory. The tumor microenvironment (TME) is an indispensable component in the process of tumor genesis and development. Due to the unique metabolism of the tumor, the pH value of TME is approximately 6.5, which is significantly lower than the pH of 7.4 in normal tissues 2-4. Therefore, antitumor treatment based on this pH difference is expected to exhibit significant efficacy and simultaneously alleviate unnecessary damage to normal tissues to a certain degree. Therefore, TME-based drug intervention provides a new strategy for antitumor therapies.

The aforementioned radiotherapy and chemotherapy exert antitumor effects by directly or indirectly generating free radicals, and reactive oxygen species (ROS) are one of the hottest topics in antitumor research *via* free radicals. Because of the elevated basal level of endogenous ROS derived from the hypermetabolism of tumor cells compared with normal cells 5, 6, tumor cells cannot effectively eliminate the accumulated exogenous ROS, which can further damage biological macromolecules such as proteins, lipids and nucleic acids *via* oxidative stress, finally resulting in tumor cell death 7-9. Due to their active chemical properties and short half-life, ROS will be severely consumed before reaching the tumor site, leading to limited antitumor effects and unnecessary damage to normal tissues. Therefore, the development of a highly effective and safe ROS source with certain tumor-targeting properties that can autonomously function without special equipment and extra energy input will undoubtedly become an important breakthrough in this research field. Recently, some well-designed hydrogen peroxide (H_2_O_2_)-generating nanosystems have displayed ideal antitumor effects 10-12. Calcium peroxide (CaO_2_) is known as solid H_2_O_2_ because of the generation of H_2_O_2_ upon reaction with H_2_O. In fact, the reaction between CaO_2_ and H_2_O varies depending on the pH value of the reaction system 13:


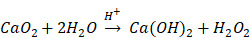



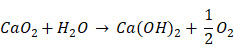


Presently, CaO_2_ is widely used in disinfection and contaminant degradation through the continuous release of H_2_O_2_ or O_2_ with significant oxidizing properties under certain conditions 14-17. In addition, CaO_2_ has been successfully applied in tissue engineering 18-20, but its antitumor application has rarely been reported. Although some studies have shown the satisfactory antitumor efficacy of CaO_2_ *via* producing O_2_ or causing calcium overload 21, 22, the antitumor mechanism of introducing oxidative stress to induce apoptosis through ROS has not been clearly studied. All of the products derived from the reaction of CaO_2_ with H_2_O are endogenous substances in organisms, ensuring excellent biocompatibility. The kinetics study of CaO_2_ reacting with H_2_O to form H_2_O_2_ under different conditions, including pH values and temperatures, has shown that the release kinetic pattern for H_2_O_2_ generation is pseudo-zero-order 13. Once CaO_2_ is exposed to H_2_O, the reaction is immediately initiated after a relatively slow dissolution process. When applied directly, CaO_2_ will be almost exhausted before arriving at the tumor site through the blood circulation without exerting the corresponding antitumor effect. Therefore, appropriate carriers are expected to effectively isolate CaO_2_ before localization at the tumor site to ensure the relatively concentrated release of ROS in the tumor tissue or inside of tumor cells.

The nanosized drug carrier can be relatively enriched in the tumor tissue through the enhanced permeability and retention (EPR) effect, showing a certain degree of tumor-targeting characteristics 23-25. Mesoporous silicon-based nanocarriers with a series of adjustable pore sizes, a large specific surface area, the excellent biocompatibility and abundant silanol groups on the surface for further functionalization are one of the most promising nanocarriers 26-29, contributing a high drug-loading capacity and various stimuli-responsive properties, including ultrasound, light, temperature, pH values, enzymes, redox responsiveness and so on 30-35. In particular, the hollow mesoporous silica nanoparticles (HMSNs) developed in recent years have a higher drug loading capacity due to their large internal cavities. Based on the pH differences between TME and normal tissues, the tumor-targeting release of loaded drugs can be achieved by coating a pH-responsive gatekeeper on the surface of HMSNs. As reported, polyacrylic acid (PAA) has a pH-dependent swelling ratio, allowing it to assist HMSNs in completing the pH-responsive release of the loaded drug when exposed to acidic TME 36. Therefore, PAA-coated HMSNs are expected to protect CaO_2_ from decomposition before the generation of ROS in TME.

**Scheme 1:**
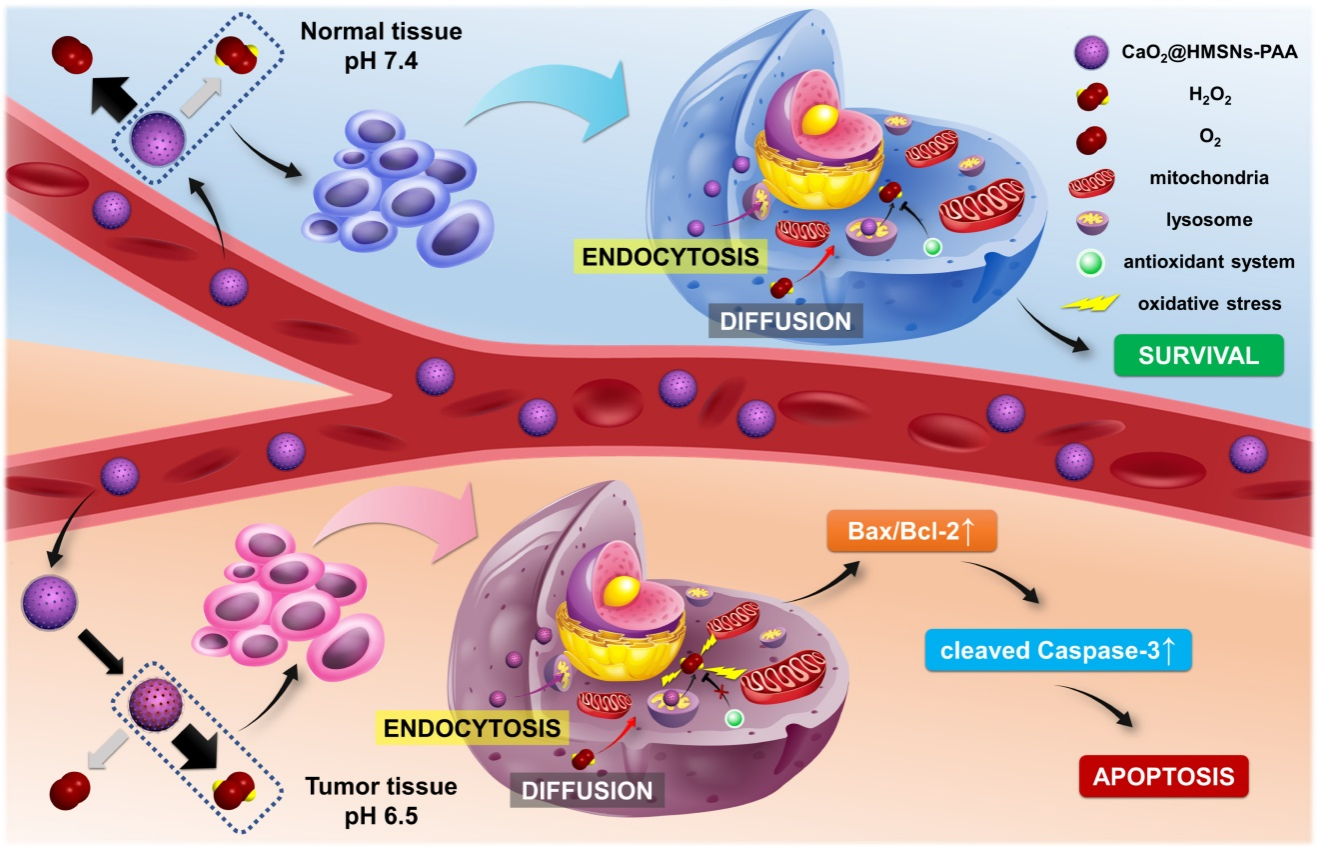
Schematic mechanism of CaO_2_@HMSNs-PAA serving as tumor environment-responsive nanoparticles for the antitumor therapy of prostate cancer. The natural pH value of the blood and normal tissues can prevent the release of H_2_O_2_ from CaO_2_@HMSNs-PAA. However, the acidic tumor environment (TME) contributes to the responsive release of CaO_2_, followed by the tendency to generate more H_2_O_2_. In addition, after intracellular uptake of CaO_2_@HMSNs-PAA by endocytosis, the more acidic environment in the lysosomes can result in the enhanced release of H_2_O_2_. Compared with normal cells, the extra exogenous ROS that has accumulated in tumor cells cannot be effectively decomposed because of the elevated basal level of ROS and the overburdened antioxidant system, consequently inducing the mitochondria-mediated apoptosis *via* remarkable oxidative stress.

Here, we establish a TME-responsive ROS-controlled-release nanosystem by loading CaO_2_ into the PAA-coated HMSNs (CaO_2_@HMSNs-PAA), which displays an ideal anti-prostate cancer effect and minimizes damages to normal tissues simultaneously. As shown in Scheme 1, in blood circulation, PAA coated on the surface of CaO_2_@HMSNs-PAA can effectively ensure the stability of the loaded CaO_2_ at pH 7.4. After reaching the tumor tissue, the acidic TME contributes to the responsive release of CaO_2_, followed by the tendency to generate more H_2_O_2_ and a small amount of oxygen. The extracellular CaO_2_@HMSNs-PAA and H_2_O_2_ enter the cell by endocytosis and diffusion, respectively 36-39. Then, CaO_2_@HMSNs-PAA localizes in lysosomes where more H_2_O_2_ will be generated under the more acidic environment with a pH value of approximately 5.0 40-42. The baseline intracellular level of ROS in tumor cells is higher than in normal cells 43, resulting in the relatively weaker ROS scavenging ability of the antioxidant system in tumor cells. Compared with the endogenous ROS at physical levels in normal cells, exogenous ROS that accumulates in tumor cells cannot be effectively decomposed, consequently leading to more obvious oxidative stress and the apoptosis of tumor cells through the mitochondria-mediated apoptosis pathway. However, CaO_2_@HMSNs-PAA exposed to normal tissues are prone to generate more oxygen and less H_2_O_2_ under neutral conditions. Afterwards, the exogenous ROS in normal cells can be effectively removed over time, thus avoiding obvious oxidative stress. Therefore, after loading into the above nanocomposite, the free CaO_2_ with no significant antitumor effects by itself can responsively release ROS in the acidic TME, thereby showing excellent antitumor efficacy and simultaneously minimizing damages on normal tissues. This concept of “efficacy-shaping nanomedicine” provides a new strategy for the treatment of prostate cancer.

## Methods

### Materials

Tetraethyl orthosilicate (TEOS), ammonium hydroxide (NH_4_OH, 25-28%), ethanol (> 99%), cetyltrimethylammonium bromide (CTAB), sodium carbonate (Na_2_CO_3_), sodium hydroxide (NaOH), hydrochloric acid (HCl, 36-38%), 3-aminopropyl triethoxysilane (APTES), toluene (> 99%), methanol (> 99%), calcium peroxide (CaO_2_) and polyacrylic acid (PAA, M.W. ~3000) were purchased from Sinopharm Chemical Reagent Co., Ltd. (China). Fluorescein isothiocyanate (FITC) was obtained from Yeasen Biotech Co., Ltd. (China). Phosphate-buffered saline (PBS, without calcium and magnesium) was acquired from Corning Incorporated (USA). All reagents were used as received without further purification. The deionized water used in all experiments was produced by a Milli-Q water purification system with a specific resistance greater than 18 MΩ.cm at 25 °C.

### Synthesis of HMSNs

The HMSNs were synthesized according to the published literature 33, 44, 45. Above all, the solid silica dioxide nanoparticles (sSiO_2_) were prepared by the modified StÖber method. Briefly, 71.4 mL of ethanol, 3.14 mL of NH_4_OH and 10 mL of H_2_O were mixed and stirred at 30 °C. After adding 6 mL of TEOS rapidly, the stirring continued for 2 h. Then, sSiO_2_ was obtained by centrifugation (12 000 rpm), washed with ethanol and H_2_O, respectively, and dried under vacuum. Subsequently, the core/shell nanoparticles (sSiO_2_@mSiO_2_) were synthesized. 0.5 g of sSiO_2_ was dispersed into 100 mL of H_2_O under ultrasonication (100 W, 40 kHz) for 30 min. Next, 0.75 g of CTAB, 150 mL of ethanol, 2.75 mL of NH_4_OH and 150 mL of H_2_O were added into the suspension of sSiO_2_. After ultrasonication (100 W, 40 kHz) for 2 h at room temperature, 1.5 mL of TEOS was introduced to the above mixture rapidly, and the reaction was maintained for another 6 h. Then, the precipitated sSiO_2_@mSiO_2_ was collected by centrifugation (12 000 rpm) and rinsed with H_2_O and ethanol. Afterwards, a selective etching approach was selected to synthesize the HMSNs. As-prepared sSiO_2_@mSiO_2_ was dispersed into 50 mL of 0.6 M Na_2_CO_3_ solution and reacted for 6 h at 80 °C. After centrifugation (12 000 rpm), the precipitate was washed with H_2_O and ethanol, and lyophilized. Finally, the template CTAB in the mesoporous channels of the as-prepared HMSNs (1 g) was removed by refluxing at 50 °C for 8 h after being dispersed into a concentrated hydrochloric acid/ethanol (1 mL/180 mL) solution mixture, followed by centrifugation (12 000 rpm) and washing with H_2_O and ethanol. The above procedure was repeated three times to obtain HMSNs without CTAB.

### Fabrication of CaO_2_@HMSNs-PAA and relevant nanocomposites

First, APTES was used to prepare the amino-functionalized HMSNs (HMSNs-NH_2_) 46, 47. 0.5 g of HMSNs were dispersed into 50 mL of toluene followed the addition of 0.4 mL of APTES. After refluxing for 10 h at 60 °C, HMSNs-NH_2_ were collected by centrifugation (12 000 rpm), washed with ethanol and H_2_O, and dried in a vacuum. Next, 0.1 g of CaO_2_ was dispersed into 15 mL of ethanol (> 99%). After adding the as-prepared 0.1 g of HMSNs-NH_2_, the mixture was stirred for 24 h at room temperature in the dark. Afterwards, CaO_2_@HMSNs-PAA was synthesized by adding 10 mL of PAA/ethanol solution (5% w/w) into the above mixture, and the stirring continued for 4 h. Finally, the product was obtained by centrifugation (12 000 rpm), washed rapidly with H_2_O and ethanol and lyophilized in the dark.

For application in control experiments, CaO_2_-loaded HMSNs (CaO_2_@HMSNs) and PAA-coated HMSNs-NH_2_ (HMSNs-PAA) were synthesized by a similar process. FITC-labeled CaO_2_@HMSNs-PAA (CaO_2_@HMSNs-PAA-FITC) used in the cellular uptake assay were prepared as follows 48: 10 mg of FITC reacted with 24 μL of APTES in 4 mL of methanol at room temperature for 24 h in the dark to form FITC-APTES. Next, 50 mg of CaO_2_@HMSNs-PAA was dispersed into the above FITC-APTES solution and the stirring continued under the same conditions for 12 h. After centrifugation (12 000 rpm), CaO_2_@HMSNs-PAA-FITC was rinsed with methanol and lyophilized in the dark. In order to evaluate the influence of the mass ratio of CaO_2_ to HMSNs on the loading efficiency (the mass of CaO_2_ loaded in CaO_2_@HMSNs / the mass of CaO_2_ used to synthesize CaO_2_@HMSNs) and the loading capacity (the mass of CaO_2_ loaded in CaO_2_@HMSNs / the mass of CaO_2_@HMSNs) during the preparation of the above nanocomposites, different mass ratios of CaO_2_ to HMSNs (0.25, 0.5, 1.0, 1.5 and 2.0) were used to prepare CaO_2_@HMSNs 49. The corresponding loading efficiencies and loading capacities were analyzed to determine the optimum mass ratio of CaO_2_ to HMSNs.

### Characterization

The transmission electron microscopy (TEM) was adopted for the morphology and element analysis on a JEOL JEM-2011 transmission electron microscope with an energy-dispersive X-ray spectroscopy (EDS) detector operated with an accelerating voltage of 200 kV. The scanning electron microscope (SEM) images and EDS mapping images were determined by a Hitachi S4800 scanning electron microscope with a working voltage of 3 kV. The low-angle X-ray diffraction (XRD) patterns were recorded on a D/max2550VB3+/PC X-ray diffractometer with Cu Kα radiation (λ = 0.15418 nm). The Fourier transform infrared (FT-IR) spectra were obtained on a Nicolet 6700 FT-IR spectrometric analyzer using KBr discs in the region of 4000-500 cm^-1^. Zeta potential and dynamic light scattering (DLS) particle size distributions were obtained using a Malvern Zetasizer Nano-ZS90 with the samples dispersed into ethanol. The nitrogen (N_2_) adsorption-desorption isothermal curve and the corresponding pore-size distribution measured by the Micromeritics Tristar 3000 system were used to detect porous structures at 77 K. The surface area (S_BET_) and pore volume (V_pore_) were determined by Brunauer-Emmett-Teller (BET) and Barrett-Joyner-Halenda (BJH) analysis, respectively. The pore-size distribution curves were obtained by BJH analysis.

### Measurement of the CaO_2_ loading capacity and release profiles

The amount of CaO_2_ incorporated in CaO_2_@HMSNs-PAA was determined by utilizing inductively coupled plasma-mass spectrometry (ICP-MS) (iCAP Q, Thermo Scientific) to detect the concentration of Ca in the suspension after the encapsulation of CaO_2_. First, 0.1 g of CaO_2_ (*m_0_*) was dispersed into 15 mL of ethanol. After the synthesis of CaO_2_@HMSNs-PAA, the precipitate was obtained by centrifugation, rapidly washed with H_2_O and ethanol, and weighed (*m_CHP_*) after lyophilization. Then, all the supernatants were collected, and HCl (2 M) of a certain volume was added. The amount of CaO_2_ in the supernatant (*m_s_*) was determined according to the volume of supernatants and the concentration of Ca measured by ICP-MS. The CaO_2_ loading capacity was calculated based on the following formula 36, 48: CaO_2_ loading capacity (%, w/w) = the mass of CaO_2_ loaded in CaO_2_@HMSNs-PAA / the mass of CaO_2_@HMSNs-PAA × 100% = (*m_0 -_ m_s_*) / *m_CHP_*× 100%. The CaO_2_ loading capacity of CaO_2_@HMSNs was evaluated with a similar procedure.

The exploration of CaO_2_ release profiles was similar to the calculation of the CaO_2_ loading capacity. First, accurately weighted CaO_2_@HMSNs and CaO_2_@HMSNs-PAA were dispersed in a certain volume of PBS with different pH values of 7.4, 6.5, and 5.0, which were used to simulate the circumstances of normal tissues, TME and lysosomes 48, 50, and stirred at room temperature. Then, the concentration of Ca in the suspension was detected by ICP-MS at a given time interval, which lasted for 2 h. Finally, the release curves were fitted based on a series of obtained concentrations of CaO_2_ to describe the release procedure 36, 48.

### Determination of the release kinetics of H_2_O_2_ from CaO_2_@HMSNs-PAA

First, 0.1 g of CaO_2_@HMSNs-PAA was dispersed in 20 mL of PBS with different pH values of 5.0, 6.5 and 7.4, before which all these PBS were degassed by reduced pressure and intermittent bubbling argon 21. To further isolate the PBS and the air, the liquid paraffin was added into the beaker 21. Then the stirring started in the dark at 37 °C. The production of H_2_O_2_ and O_2_ in the suspension was measured by an H_2_O_2_ assay kit (Nanjing Jiancheng Bioengineering Institute) and an Unisense oxygen microsensor, respectively, at given time points 12, 21, 49, 51. Finally, the release curves were plotted based on a series of obtained concentrations to describe the release kinetics of H_2_O_2_ and O_2_ from CaO_2_@HMSNs-PAA.

### Detection of the dispersibility and stability of CaO_2_@HMSNs-PAA

1 mg of CaO_2_@HMSNs-PAA was dispersed in 10 mL of H_2_O, PBS and Kaighn's Modification of Ham's F-12 (F-12K) medium + 10% fetal bovine serum (FBS) at 37 °C, then the DLS particle size distributions and polydispersity index (PDI) on Day 0, Day 1 and Day 3 were obtained by a Malvern Zetasizer Nano-ZS90 51-54.

### Cell culture

The human prostate cancer cell line PC-3 and the human prostate epithelial cell line RWPE-1 were purchased from the Cell Bank of Type Culture Collection of the Chinese Academy of Sciences (Shanghai, China), where it has passed conventional evaluations of cell line quality control, including DNA fingerprinting, isozymes, mycoplasma, morphology and so on. The cell line was cultured in F-12K medium (Gibco) containing 2 mM L-glutamine and 2500 mg/L sodium bicarbonate, supplemented with 10% FBS (Gibco), 100 U/mL penicillin and 100 μg/mL streptomycin (Gibco). The cell line was incubated in an incubator (Heracell 150i, Thermo Scientific) at 37 °C under an atmosphere of 5.0% CO_2_ and 90% relative humidity and was subcultured for subsequent experiments by the addition of 0.25% trypsin (Gibco), 10% FBS, and fresh F-12K medium. All cell experiments were performed with cells in the logarithmic growth phase.

### Intracellular uptake assay

A Leica TCS SP8 confocal laser scanning microscopy (CLSM) was employed to evaluate the intracellular uptake and endocytosed distribution of CaO_2_@HMSNs-PAA, according to previous reports 41. First, PC-3 cells were seeded into 24-well plates with cover glass for growth at a density of 1 × 10^5^ cells per well at 37 °C overnight. Then, the cells were incubated with the CaO_2_@HMSNs-PAA-FITC/F-12K medium solution (50 μg/mL, 1 mL/well) for 0.5 h, 1 h and 3 h at 37 °C in the dark, followed by rinsing with PBS to remove the residual nanoparticles after the medium was discarded. Subsequently, the LysoTracker Red (Beyotime)/F-12K medium solution (50 nM, 0.5 mL/well) was supplemented to the wells followed by incubation at 37 °C for 1 h in the dark to label lysosomes. After discarding the above lysosome-staining solution and washing the cells with PBS, the 4,6-diamidino-2-phenylindole (DAPI) (KeyGEN)/methanol solution (10%, 0.5 mL/well) was added and incubated for 15 min at 37 °C in the dark to fix the cells and stain the nuclei. Afterwards, the above working solution was discarded. Then, the Trypan blue (Yeasen)/PBS solution (2 mg/mL, 0.5 mL/well) was introduced to quench the intracellular fluorescence at 37 °C for 2 min in the dark 48. Subsequently, the cells were washed with PBS and methanol, respectively, and the buffer solution included in the above DAPI detection kit was added to the wells. Finally, after slide-making, CLSM was adopted to observe the intracellular uptake and subcellular location of CaO_2_@HMSNs-PAA. The ImageJ software (Ver.1.52i) was utilized to analyze the intracellular fluorescence intensities of FITC in 0.5 h, 1 h and 3 h after incubation. Then, the Pearson's correlation analysis between the uptake and the incubation time was implemented based on the above data.

### *In vitro* cytotoxicity assay

The CCK-8 cell proliferation assay was conducted to evaluate the cytotoxicities of HMSNs, HMSNs-PAA, CaO_2_, CaO_2_@HMSNs and CaO_2_@HMSNs-PAA against PC-3 cells. The PC-3 cells were planted into 96-well plates at a density of 1 × 10^4^ cells per well in 100 μL of F-12K medium and were incubated at 37 °C overnight. Then, the culture medium was replaced with 100 μL of fresh F-12K medium containing serial dilutions of CaO_2_ or as-prepared nanoparticles, and the cells were further incubated for 24 h at 37 °C in the dark. Cells cultured in F-12K medium without any materials were adopted as the negative control group. To simulate the pH values of the neutral normal tissues and the acidic TME, the pH value of the F-12K medium used in the experiments was adjusted to 7.4 or 6.5 by the addition of HCl (1 M) 50. The final concentrations of CaO_2_ itself or loaded in CaO_2_@HMSNs and CaO_2_@HMSNs-PAA dispersed in F-12K medium in the different treatment groups were set as 8 μg/mL, 16 μg/mL, 32 μg/mL and 64 μg/mL. Similarly, the serial concentrations of HMSNs and HMSNs-PAA suspended in F-12K medium were 25 μg/mL, 50 μg/mL, 100 μg/mL and 200 μg/mL. After washing the cells with PBS, 10 μL of CCK-8 solution (Yeasen) mixed with 90 μL of F-12K medium was added to each well, and the incubation lasted for another 2 h. Finally, the absorbance values of relevant wells at the wavelength of 450 nm were measured by a hybrid multimode microplate reader (Synergy H4+, BioTek). Cell proliferation was calculated according to the following formula: cell proliferation (%) = (A_test_-A_blank_) / (A_control_-A_blank_) × 100%, where A_test_ and A_control_ were the absorbance values of the groups treated with CaO_2_ or nanoparticles and the negative control group, respectively, and A_blank_ was the absorbance value of the CCK-8 reagent/F-12K medium at 450 nm.

Similarly, in order to confirm the cytotoxicity of CaO_2_@HMSNs-PAA against normal prostate epithelial cells under a simulated normal body fluid environment, the CCK-8 assay was also conducted after 24 h of incubation of RWPE-1 cells with HMSNs, HMSNs-PAA, CaO_2_, CaO_2_@HMSNs and CaO_2_@HMSNs-PAA in PEpiCM medium (pH 7.4) with the equivalent CaO_2_ concentration of 16 μg/mL. In addition, to further determine that the cytotoxicity of CaO_2_@HMSNs-PAA mainly derived from H_2_O_2_ rather than Ca^2+^, PC-3 cells were incubated with catalase (CAT) (200 U/mL), CaO_2_@HMSNs-PAA (16 μg/mL, calculated by CaO_2_) and CaO_2_@HMSNs-PAA (16 μg/mL, calculated by CaO_2_) + CAT (200 U/mL) under the simulated TME condition (pH 6.5) 55, 56, and the CCK-8 assay was utilized to determine the cytotoxicity due to the above 24 h of incubation.

### Observation of intracellular ROS

To detect the intracellular ROS generated by the as-prepared nanocomposites, PC-3 cells were inoculated into 6-well plates at a cell density of 5 × 10^5^ cells per well at 37 °C overnight. Afterwards, the cells were incubated with CaO_2_, CaO_2_@HMSNs and CaO_2_@HMSNs-PAA in F-12K medium with an equivalent CaO_2_ concentration of 16 μg/mL (2 mL/well) at 37 °C for 4 h in the dark. Simultaneously, HMSNs and HMSNs-PAA with an equivalent amount of relevant constituent with that in the CaO_2_@HMSNs and CaO_2_@HMSNs-PAA were also dispersed into F-12K medium for cell incubation in relevant groups. As mentioned above, the pH value of the F-12K medium used to mix with CaO_2_ and the as-prepared nanoparticles were adjusted to 7.4 or 6.5 to simulate the neutral normal tissue environment and the acidic TME. Meanwhile, cells incubated with F-12K medium without CaO_2_ or nanocomposites were used as the negative control. After discarding the medium, the residual materials were removed by washing the cells with PBS. Then, the 2',7'-dichlorodihydrofluorescein diacetate (DCFH-DA) (Beyotime)/F-12K medium solution (10 μM, 2 mL/well) was introduced into the wells, and the cells were incubated for another 30 min. After washing the cells with PBS, a Leica DMI3000B fluorescence microscopy was adopted to evaluate the intracellular fluorescent intensity. Furthermore, the ImageJ software (Ver.1.52i) was adopted to analyze the intracellular fluorescence intensities of 2',7'-dichlorofluorescein (DCF) in each group.

### Analysis of the *in vitro* apoptosis

An Annexin V-FITC/propidium iodide (PI)-based apoptosis detection kit (Yeasen) and flow cytometry (FCM) were employed to detect PC-3 cells apoptosis induced by the as-prepared nanoparticles. Briefly, PC-3 cells were seeded into 6-well plates at a density of 5 × 10^5^ cells per well in 2 mL of F-12K medium at 37 °C overnight. Similar to the evaluation of intracellular ROS, the cells were exposed to different materials dispersed in F-12K medium at a pH value of 7.4 or 6.5 at 37 °C for 24 h in the dark. For comparison, the cells incubated with F-12K medium without CaO_2_ or nanoparticles were set as the negative control group. Then, the medium containing the suspended cells was collected. After washing with PBS and digesting with 0.25% trypsin, the cells were obtained by centrifugation and resuspended in 100 μL of binding buffer. Next, Annexin V-FITC and PI were further introduced following the manufacturer's instructions. Subsequently, the cells were stained at room temperature for 15 min in the dark, and 400 μL of binding buffer was supplemented. Finally, all these samples were analyzed by a CytoFLEX flow cytometer (Beckman Coulter) to record the apoptosis rate of the different groups.

### Western blot analysis

Similar to the above cellular apoptosis assays, PC-3 cells were seeded into 6-well plates at a density of 5 × 10^5^ cells per well in 2 mL of F-12K medium at 37 °C overnight. Then, these cells were exposed to different materials dispersed in F-12K medium with a pH value of 7.4 or 6.5 at 37 °C in the dark. Moreover, the cells incubated with F-12K medium without CaO_2_ or nanoparticles were set as the negative control group. After incubation of 24 h, the cells in different groups were lysed by radioimmunoprecipitation assay (RIPA) lysis buffer (Beyotime) to obtain the total proteins. Protein concentrations were determined by a BCA protein assay kit (Beyotime) according to the manufacturer's instructions. Subsequently, 30 μg of protein were separated by electrophoresis on 10% SDS-polyacrylamide gels (Beyotime), followed by transferring to polyvinylidene difluoride (PVDF) membranes (Millipore). After blocking by 5% skim milk (Beyotime), the membranes were incubated overnight at 4 °C with primary antibodies against β-actin (1:1000, ABclonal), Bax (1:1000, Cell Signaling Technology), Bcl-2 (1:1000, Cell Signaling Technology) and cleaved Caspase-3 (1:1000, Cell Signaling Technology). After washing with Tris-buffered saline/Tween-20 (TBST) solution, the membranes were incubated with the horseradish peroxidase (HRP)-conjugated secondary antibody (1:1000, Beyotime) at room temperature for 1 h. Then, the blots were developed by an enhanced chemiluminescence (ECL) kit (Beyotime) and visualized by a ChemiQ 4600mini imaging system (Shanghai Bioshine Science Instruments Co., Ltd.). Finally, the band densities were analyzed by the ImageJ software (Ver.1.52i).

### Animals

Male BALB/c mice and male BALB/c nude mice aged 5 weeks were purchased from Shanghai SLAC Laboratory Animal Co., Ltd. (China). All animal procedures were carried out according to the protocols approved by the Ethics Committee of Xinhua Hospital Affiliated to Shanghai Jiao Tong University School of Medicine. All *in vivo* experiments were performed under the guidelines of the National Institute of Health Guide for the Care and Use of Laboratory Animals and the Animal Care and Use Committee of Shanghai Jiao Tong University School of Medicine. All the mice were randomly allocated, and no more than five were group-housed per cage with a reversed 12 h light/dark cycle at a temperature of 25 ± 2 °C and relative humidity of 70 ± 5%.

### Evaluation of the *in vivo* biosafety

Healthy male BALB/c mice aged 5 weeks were randomly separated into four groups (n = 5) based on the intravenous dose of CaO_2_@HMSNs-PAA dispersed in PBS (0 mg/kg, 10 mg/kg, 20 mg/kg and 40 mg/kg). After injection of the as-prepared nanoparticles through the tail vein, the body weights of all the mice were recorded every 3 days to observe the potential influence of the materials on bodily functions. After 30 days of measuring, all the mice were sacrificed by cervical dislocation. Another 48 male BALB/c mice aged 5 weeks were assigned into four groups (n = 12) at random, followed by treatment with a similar process. Three mice in each group were randomly sacrificed on day 0, day 1, day 7 and day 30 after intravenous injection of CaO_2_@HMSNs-PAA. Then, the major organs of the mice (the heart, liver, spleen, lung and kidney) and blood were collected for histological and blood analysis. The liver function indicators including alanine aminotransferase (ALT), aspartate aminotransferase (AST) and alkaline phosphatase (ALP), renal function-related blood urea nitrogen (BUN) and creatinine (CREA), along with the blood panel parameters including white blood cells (WBC) count, red blood cells (RBC) count, hemoglobin (HGB), hematocrit (HCT), mean corpuscular volume (MCV), mean corpuscular hemoglobin (MCH) and mean corpuscular hemoglobin concentration (MCHC) were measured by a biochemical analyzer (AU480, Beckman Coulter) and a blood cell analyzer (BC-5390, Mindray), respectively.

### Tumor models

A subcutaneous PC-3 xenografted tumor model was used in this study. Healthy male BALB/c nude mice aged 5 weeks were subcutaneously inoculated in the right hind limb space with 1 × 10^6^ PC-3 cells suspended in 100 μL of F-12K medium without FBS. Then, the biodistribution assay and *in vivo* antitumor evaluation and of CaO_2_@HMSNs-PAA were performed when the tumor volume reached approximately 100 mm^3^.

### Biodistribution analysis

The above PC-3 tumor model was adopted to assess the biodistribution of CaO_2_@HMSNs-PAA after intravenous administration. After the tumor volumes reached about 100 mm^3^, twelve PC-3 xenografted tumor-bearing mice were injected with the CaO_2_@HMSNs-PAA/PBS solution (40 mg/kg) *via* the tail vein. Subsequently, all the mice were randomly assigned into four groups (n = 3) to be sacrificed separately by cervical dislocation at 3 h, 6 h, 12 h and 24 h post-injection. Then, the tumor and major organs (the heart, liver, spleen, lung and kidney) of each mouse were dissected, rinsed with PBS, weighed (*m_organ_*, g) and homogenized, followed by digestion with aqua regia. Afterwards, the quantitative analysis of the element Si was determined by ICP-MS. Finally, the percent injected dose per gram of the organ (D, % ID/g) of Si was calculated according to the following equation 57: D (% ID/g) = (*m* / *m_organ_*) / *m_ID_*× 100%, where *m* (μg) is the mass of Si in the measured organ and *m_ID_* (μg) represents the total injected dose of Si.

### Evaluation of the *in vivo* antitumor effect

When the tumor volumes reached approximately 100 mm^3^ (designed as day 0), the PC-3 tumor-bearing BALB/c nude mice were randomly allocated into six groups (n = 5) followed by intravenous administration of PBS (10 μL/g) only (as the control group) or intravenous injection containing CaO_2_@HMSNs-PAA (40 mg/kg) or an equivalent amount of CaO_2_, CaO_2_@HMSNs, HMSNs and HMSNs-PAA. Then, the maximum length (L) and maximum width (W) of all the tumors and body weights of all the mice were measured every 2 days with a digital caliper and an electronic balance, respectively. The tumor volume (V) was calculated as V = L × W^2^ / 2 57, and the relative tumor volume (V / V_0_) was normalized to the initial volume (V_0_) 57. The injection of the materials was conducted only once. In consideration of the care regulations, all tumor-bearing mice were sacrificed by cervical dislocation for anatomical and histopathological analysis on day 14 after injection. Kaplan-Meier survival analysis was conducted in the above PC-3 tumor-bearing BALB/c nude mice, with the definition of survival as the tumor volume (V) failed to exceed two times of the initial volume (V_0_).

To further explore the mechanism of the antitumor effect from the as-prepared nanoparticles, another eighteen PC-3 tumor-bearing BALB/c nude mice were also assigned into six groups randomly with a similar procedure (n = 3). On day 2 post-administration of the respective materials, all tumors were collected after the mice were sacrificed by cervical dislocation. Then, the expression of Bax, Bcl-2 and cleaved Caspase-3 in the tumor was detected by immunohistochemical analysis. Simultaneously, the TUNEL assay was performed to evaluate the apoptosis of tumor cells in these tumor tissues.

### Histology and immunohistochemistry

Tumors and major organs (the heart, liver, spleen, lung and kidney) were harvested at the given time, followed by fixation in a 4% polyoxymethylene solution (Biossci). After embedded in paraffin (Leica), the sections were stained with a hematoxylin and eosin (H&E) staining kit (Beyotime) to evaluate changes in tissue structure. Meanwhile, immunohistochemistry was performed to analyze the expression of some mitochondria-mediated apoptosis markers (Bax, Bcl-2 and cleaved Caspase-3) in tumor tissues stained with primary antibodies against Bax (1:100, Cell Signaling Technology), Bcl-2 (1:100, Abcam) and cleaved Caspase-3 (1:100, Cell Signaling Technology). Finally, all the images were obtained by an upright microscope (BX51, Olympus).

### TUNEL assay

TUNEL apoptosis assay kit (Beyotime) and DAPI detection kit (KeyGEN) were utilized to evaluate the apoptosis of tumor cells in the above tumor tissues based on the manufacturer's instructions. Then, an Olympus BX51 upright microscope was adopted to obtain the fluorescence images.

### Statistical analysis

Statistical comparison was conducted by analysis of variance (ANOVA) with SPSS 25 when necessary, and all data are presented as the mean ± SD unless otherwise stated. P values of < 0.05 were considered statistically significant.

## Results and Discussion

### Preparation and characterization of CaO_2_@HMSNs-PAA

**Scheme 2:**
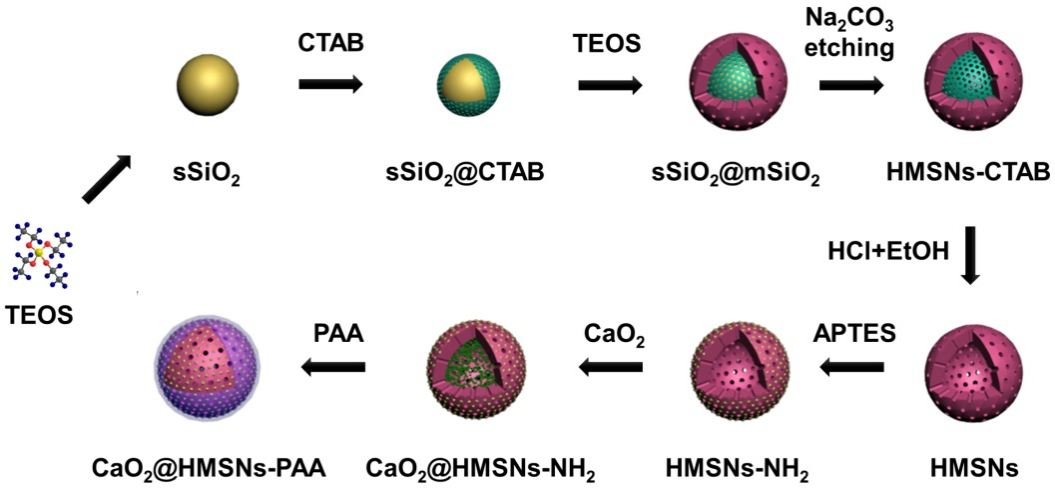
Schematic diagram of the preparation of CaO_2_@HMSNs-PAA. HMSNs were obtained through a structural difference-based selective etching approach (TEOS, tetraethyl orthosilicate; CTAB, cetyltrimethylammonium bromide; EtOH, ethanol). Then, 3-aminopropyl triethoxysilane (APTES) was conjugated onto the surface of HMSNs to form the amino-functionalized HMSNs (HMSNs-NH_2_). Finally, CaO_2_@HMSNs-PAA was fabricated by CaO_2_ loading and polyacrylic acid (PAA) coating.

As illustrated in Scheme 2, four main steps were involved in the whole preparation of CaO_2_@HMSNs-PAA. First, HMSNs were obtained through a structural difference-based selective etching approach. Then, APTES was conjugated onto the surface of HMSNs to form amino-functionalized HMSNs (HMSNs-NH_2_). Next, CaO_2_ was loaded into the channels and cavities of HMSNs-NH_2_ (forming CaO_2_@HSMNs-NH_2_) by stirring. Finally, PAA was coated onto the amino groups of CaO_2_@HMSNs-NH_2_ (forming CaO_2_@HMSNs-PAA) as the gatekeeper to realize the pH-responsive release of CaO_2_. As shown in Figure S1, at first, both the loading capacity and the loading efficiency raise as the mass ratio of CaO_2_ to HMSNs increased. However, the loading capacity was no longer elevate obviously and the loading efficiency decreased significantly when the mass ratio of CaO_2_ to HMSNs was more than 1.0, so this optimum mass ratio was adopted to improve efficiency and reduce cost in the synthesis of all the CaO_2_-loaded nanocomposites.

**Figure 1:**
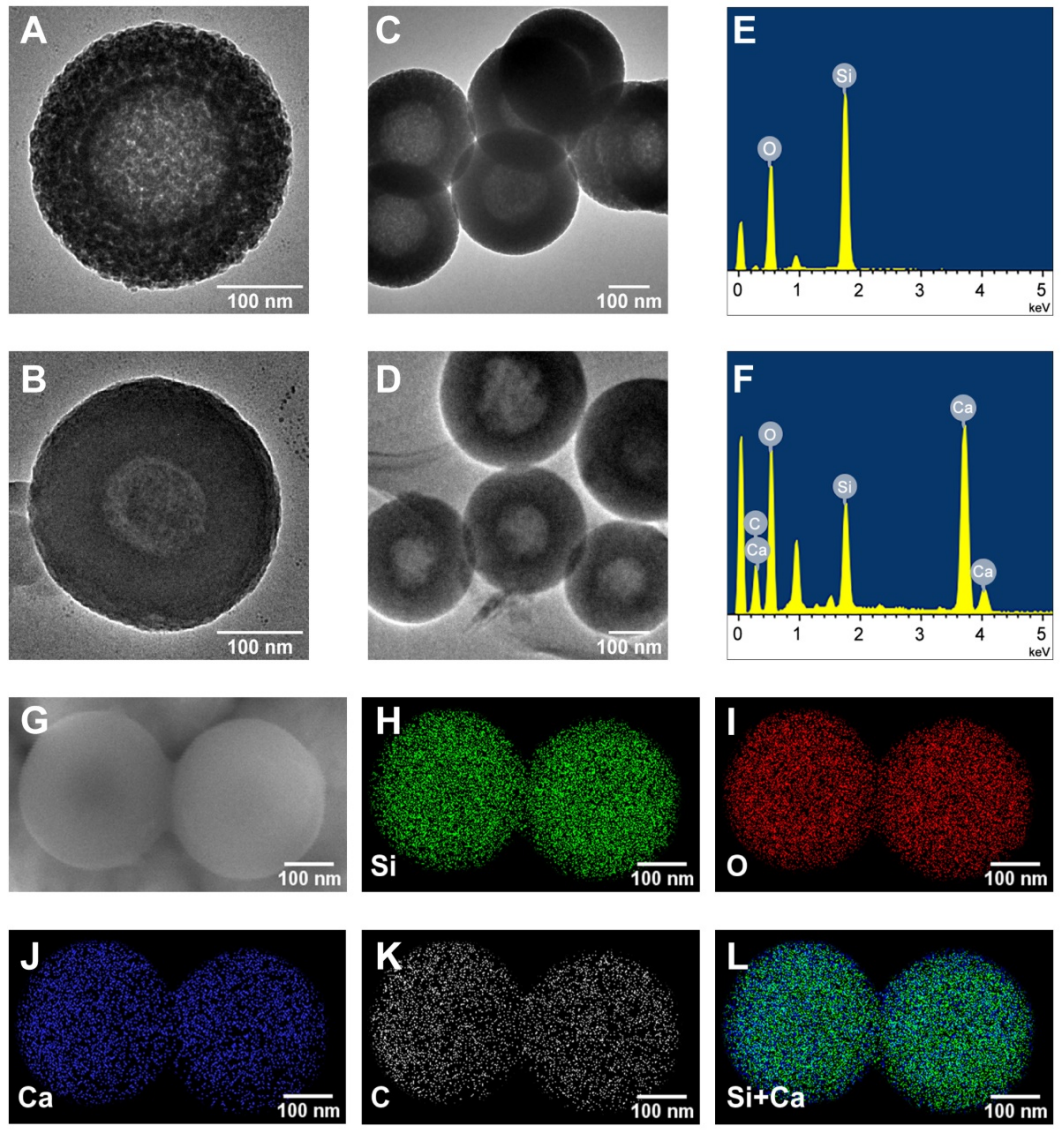
** Morphology and elemental composition of HMSNs and CaO_2_@HMSNs-PAA.** (**A-D**) Transmission electron microscopy (TEM) images of HMSNs (A, C) and CaO_2_@HMSNs-PAA (CHP) (B, D). (**E-F**) Energy-dispersive X-ray spectroscopy (EDS) spectra of HMSNs (E) and CHP (F). (**G**) Scanning electron microscope (SEM) image of CHP. (**H-L**) EDS mapping images of Si (H), O (I), Ca (J), C (K) and Si+Ca (L) in CHP. Scale bars, 100 nm.

**Figure 2:**
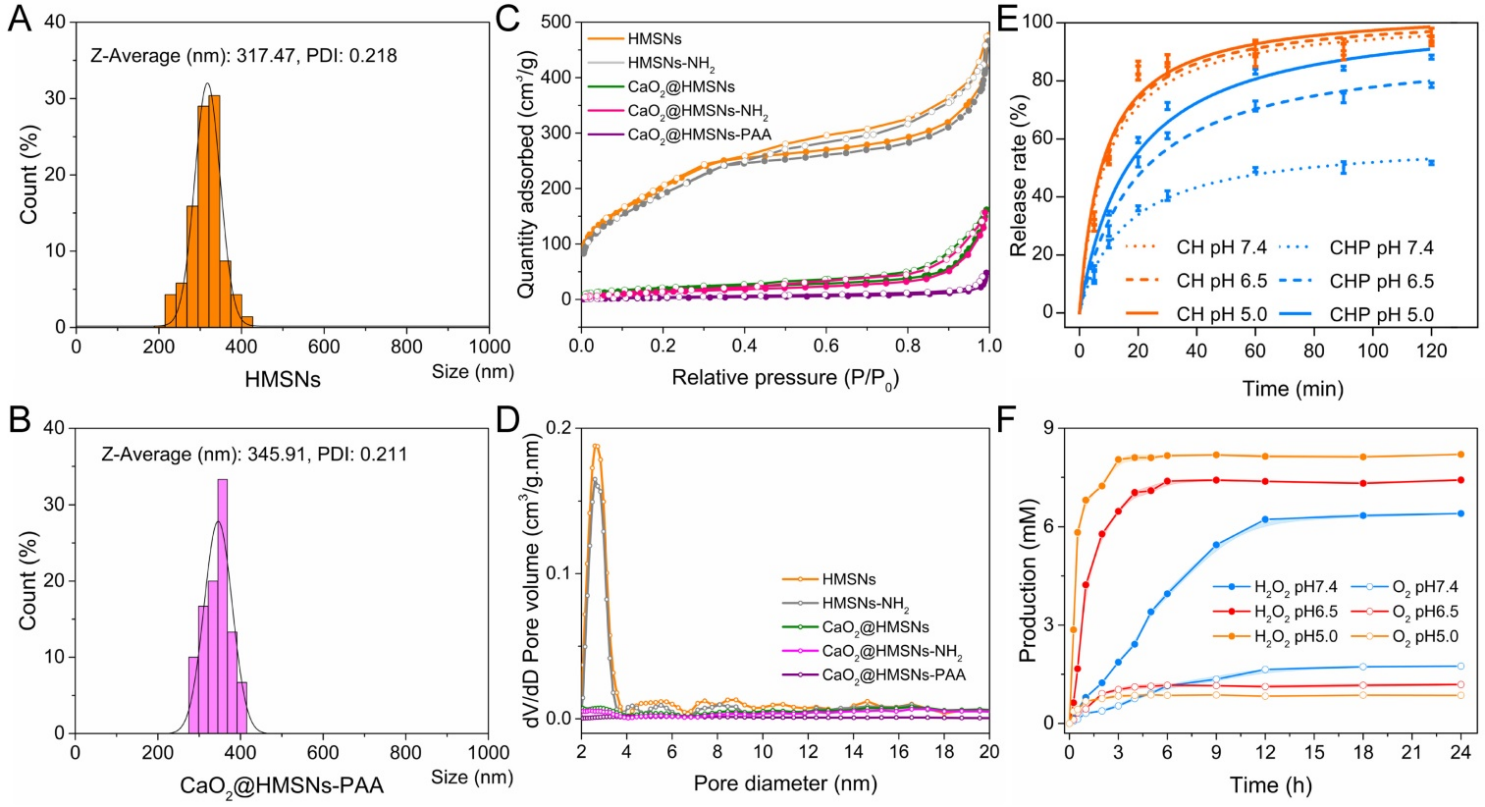
** Further characterization and responsive release profiles of CaO_2_@HMSNs-PAA.** (**A-B**) Dynamic light scattering (DLS) particle size distributions of HMSNS (A) and CaO_2_@HMSNs-PAA (B). (**C-D**) N_2_ adsorption-desorption isotherms (C) and pore-size distribution curves (D) of the as-prepared nanocomposites. (**E**) CaO_2_ release profiles from CaO_2_@HMSNs (CH) and CaO_2_@HMSNs-PAA (CHP) in phosphate-buffered saline (PBS) with different pH values. (**F**) H_2_O_2_ and O_2_ release profiles from CaO_2_@HMSNs-PAA in PBS with different pH values at 37 °C.

The TEM was utilized to observe the morphologies of HMSNs and CaO_2_@HMSNs-PAA (Figure 1A-D). HMSNs were well-dispersed spheres with an average diameter of approximately 300 nm, which were hollow with a mesoporous shell of about 75 nm, consistent with the DLS particle size distribution results (Figure 2A). The surface of CaO_2_@HMSNs-PAA became rougher due to the coating of PAA as a gatekeeper to control the release of CaO_2_. Compared with HMSNs, the inconspicuous mesoporous structure and hollow core of both CaO_2_@HMSNs and CaO_2_@HMSNs-PAA indicated the successful loading of CaO_2_ in the channels and cavities of HMSNs (Figure S2 and Figure 1B). As shown in Figure S3, the inconspicuous mesoporous structure of CaO_2_@HMSNs-PAA became more evident under the environment with the lower pH value, ensuring the ideal pH-responsiveness for the release of CaO_2_. Moreover, compared with the elemental analysis of HMSNs, the EDS spectrum of CaO_2_@HMSNs-PAA showed extra intensive signals from the elements Ca and C, indicating the successful CaO_2_ loading and PAA coating (Figure 1E-F). Furthermore, the SEM images and EDS mapping images in Figure 1G-L further confirmed the successful synthesis of CaO_2_@HMSNs-PAA 49, 51, 52, 58.

As shown in Figure 2A-B, the slightly increased size of CaO_2_@HMSNs-PAA could also be attributed to the PAA coating. Figure S4 shows that the results of low-angle XRD were also consistent with the TEM images. HMSNs exhibited a diffraction peak at 2θ = ca. 2.3386° because of the ordered mesoporous structure. However, due to the pore-filling effect caused by CaO_2_ loading and PAA coating, the diffraction peaks almost disappeared in CaO_2_@HMSNs and CaO_2_@HMSNs-PAA (Figure S4).

The FT-IR spectra were further adopted to confirm the successful CaO_2_ loading and PAA coating during the synthesis of CaO_2_@HMSNs-PAA. As shown in Figure S5, HMSNs exhibited four characteristic peaks: the peaks at 1226 cm^-1^ and 1050 cm^-1^ were attributed to the asymmetric stretching vibrations of Si-O-Si; the symmetric stretching vibration of Si-O-Si and the stretching vibration of Si-OH appeared at 793 cm^-1^ and 958 cm^-1^, respectively 50. Because of the amino functionalization by APTES, HMSNs-NH_2_ displayed new peaks at 2933 cm^-1^, 1555 cm^-1^ and 695 cm^-1^, which originated from C-H asymmetric stretching, N-H bending vibrations and the CH_2_ rocking vibration of Si-CH_2_R 59. Due to the loading of CaO_2_, a new peak at 875 cm^-1^ in CaO_2_@HMSNs could be designated to the O-O bridge of CaO_2_ 60. Compared with HMSNs-NH_2_ and CaO_2_, the stretching vibration of amide I and amide II resulted in new peaks at 1646 cm^-1^ and 1543 cm^-1^ in CaO_2_@HMSNs-PAA 61, indicating the expected coating with PAA by the cross-link between CaO_2_-loaded HMSNs-NH_2_ and PAA 62.

The N_2_ adsorption-desorption isotherm curve and corresponding pore-size distribution were chosen to further manifest the effects of CaO_2_ loading and PAA coating during the preparation of CaO_2_@HMSNs-PAA. HMSNs displayed a typical Langmuir type IV isotherm with a type H_2_ hysteresis loop (Figure 2C), demonstrating the excellent mesoporous structure 36. The relative pressure (P/P_0_) decreased in both CaO_2_@HMSNs and CaO_2_@MSNs-PAA, and almost no P/P_0_ leap could be detected in CaO_2_@HMSNs-PAA, which proved the successful loading of CaO_2_ and coating of PAA. BET measurements revealed that the S_BET_ and the V_pore_ of HMSNs were 779.37 m^2^/g and 0.66 cm^3^/g, respectively, which sharply decreased to 69.70 m^2^/g and 0.24 cm^3^/g after CaO_2_ loading. Corresponding parameters of CaO_2_@HMSNs-PAA were further reduced to 12.44 m^2^/g and 0.06 cm^3^/g, respectively. Analogously, compared with the average HMSNs pore diameter of 3.37 nm, the pore size dropped significantly in CaO_2_@HMSNs and then could hardly be detected in CaO_2_@HMSNs-PAA (Figure 2D), exhibiting an evident pore-filling effect that was accredited to CaO_2_ loading and PAA coating.

The potential changes before and after CaO_2_ loading and surface modification were determined by zeta potential analysis. As shown in Figure S6, HMSNs manifested a negative potential of -21.53 mV when dispersed in ethanol, while HMSNs-NH_2_ exhibited a positive potential of 17.07 mV because of the amino functionalization by conjugation of APTES onto HMSNs. Then, the zeta potential was measured to be -14.67 mV after CaO_2_ loading and further decreased to -28.37 mV after additional PAA coating. All these above results of characterization proved the successful synthesis of CaO_2_@HMSNs-PAA.

### CaO_2_ loading capacity and release profiles

During the synthesis of CaO_2_@HMSNs and CaO_2_@HMSNs-PAA, CaO_2_ was loaded into the mesoporous channels and inner cavities of the as-prepared HMSNs through a simple molecular diffusion in the CaO_2_/ethanol solution. Therefore, according to the concentration of Ca in the supernatant after encapsulation determined by inductively coupled plasma (ICP) element analysis 63, the loading content of CaO_2_ in CaO_2_@HMSNs and CaO_2_@HMSNs-PAA could be calculated. Due to the slight leakage of CaO_2_ and the addition of PAA during PAA coating, the loading capacity of CaO_2_ in CaO_2_@HMSNs-PAA (20.34%) was slightly lower than that of CaO_2_@HMSNs (26.03%).

Similarly, the evaluation of the CaO_2_ release profiles was also based on the concentrations of Ca in suspensions detected by ICP-MS. Figure 2E shows the release profiles of CaO_2_ from CaO_2_@HMSNs and CaO_2_@HMSNs-PAA in PBS (without calcium and magnesium) with pH values of 7.4, 6.5 and 5.0. These PBS were chosen to mimic the environment of normal tissues/blood (pH 7.4), TME (pH 6.5) and lysosomes (pH 5.0) 41, 50. The release of CaO_2_ from the two nanocomposites presented a similar sustained pattern with different release rates. The uncoated pores in HMSNs had no confinement effect for releasing CaO_2_ from mesoporous channels because the dispersed CaO_2_ (about 1.03 nm, Figure S7) was much smaller in size than the pores in HMSNs (about 3.37 nm in size). Therefore, the release of CaO_2_ from CaO_2_@HMSNs was unconfined and analogous in PBS with different pH values. At 30 min, the released amount was 84.12% at pH 7.4, 87.02% at pH 6.5 and 87.72% at pH 5.0. After 120 min, the release rate reached 93.82% at pH 7.4, 94.17% at pH 6.5 and 96.02% at pH 5.0. However, due to the PAA coating, the CaO_2_ release pattern of CaO_2_@HMSNs-PAA was quite different from that of CaO_2_@HMSNs and changed with the pH value of PBS. This pH-responsive release derived from the distinct flexibility and swelling behavior of PAA under conditions with different pH values. After the pH value of PBS was adjusted to 7.4, the coated PAA layer could block the pores in HMSNs effectively and hinder the CaO_2_ releasing efficiently, which could also increase the stability of CaO_2_. Upon exposure to the simulated acidic TME (pH 6.5), the swelling ratio of PAA increased conspicuously, which partially liberated the pore outlets, and the osmotic pressure ensured more H_2_O to permeate into mesoporous channels of HMSN from PBS 36, leading to more CaO_2_ release. Therefore, compared with the release rate of CaO_2_ from CaO_2_@HMSNs-PAA at pH 7.4 after 30 min (40.42%), the release rate was much higher at pH 6.5 (61.08%). While the pH value was further reduced to 5.0, the HMSNs pores were almost open due to the pH-responsive characteristics of PAA, resulting in a faster release of CaO_2_. Therefore, the release rate of CaO_2_ from CaO_2_@HMSNs-PAA reached 71.31% at pH 5.0 after 30 min. As the exposure time extended to 120 min, the release amount of CaO_2_ from CaO_2_@HMSNs-PAA slowly rose to 51.73% at pH 7.4, 78.49% at pH 6.5 and 88.21% at pH 5.0. Although this *in vitro* release experiment is difficult to simulate the actual *in vivo* release process of CaO_2_ completely, especially the stirring operation may cause CaO_2_ release faster than *in vivo*, it still provides considerable evidence for the *in vivo* responsive release of CaO_2_. Based on the above results, CaO_2_@HMSNs-PAA with a pH-responsive controlled release property could release less CaO_2_ in normal tissues and minimize the unnecessary loss of CaO_2_ during blood circulation to deliver more CaO_2_ to the tumor site, which could increase the antitumor efficacy of the ROS originating from CaO_2_ and mitigate the adverse effects to normal tissues.

### Release kinetics of H_2_O_2_ from CaO_2_@HMSNs-PAA

As mentioned above, there has been a systematic report on the generation kinetics of H_2_O_2_ and O_2_ from the reaction between CaO_2_ and H_2_O under different conditions 13, which indicated that H_2_O_2_ could be generated from CaO_2_ with the pH-responsiveness. In addition, the results of the CaO_2_ release profiles in this study also revealed the pH-responsive release of CaO_2_ from CaO_2_@HMSNs-PAA. In order to further confirm the pH-responsive feature of CaO_2_@HMSNs-PAA in releasing H_2_O_2_, the release kinetics of H_2_O_2_ and O_2_ from CaO_2_@HMSNs-PAA were evaluated under the environment with different pH values. As shown in Figure 2F, CaO_2_@HMSNs-PAA could rapidly release more H_2_O_2_ and less O_2_ under the simulated circumstances of TME and lysosomes, while less H_2_O_2_ and more O_2_ were slowly released from CaO_2_@HMSNs-PAA under the simulated circumstance of normal tissues. So, due to the pH-responsive CaO_2_ release profiles and the pH-responsive H_2_O_2_ generation from CaO_2_, CaO_2_@HMSNs-PAA could release H_2_O_2_ in a pH-responsive way, laying a scientific foundation for its pH-responsive antitumor effects.

### Dispersibility and stability of CaO_2_@HMSNs-PAA

CaO_2_@HMSNs-PAA was well dispersed in all the three different solvents including H_2_O, PBS and F-12K medium + 10% FBS, and no significant change in DLS particle size distribution and PDI was detected during the observing period (Figure S8), demonstrating the ideal dispersibility and stability of CaO_2_@HMSNs-PAA which was conducive to exerting the promising antitumor efficacy.

### Intracellular uptake of CaO_2_@HMSNs-PAA

**Figure 3:**
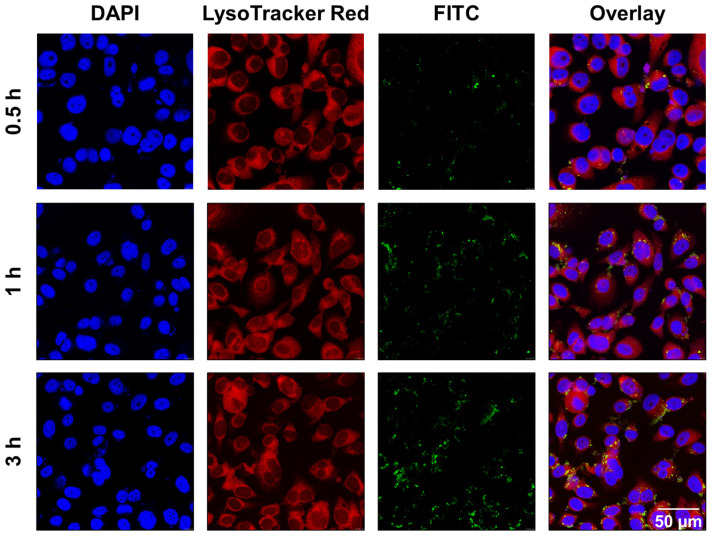
** Intracellular uptake of CaO_2_@HMSNs-PAA in PC-3 cells.** Confocal laser scanning microscopy (CLSM) images of PC-3 cells describing the intracellular uptake of FITC-labeling CaO_2_@HMSNs-PAA (green) with the nuclei stained by DAPI (blue) and lysosomes labeled by LysoTracker Red (red) after incubation for 0.5 h, 1 h and 3 h. Scale bar, 50 µm.

According to published literature, the main cellular uptake pattern of nanomaterials with diameters below 500 nm are usually endocytosis 64. The size of the as-prepared CaO_2_@HMSNs-PAA is approximately 300 nm, so it supposedly enters cells *via* an endocytic pathway 36. Lysosomes are regarded as the terminals of endocytosis, in which most of the internalized macromolecules are degraded by a variety of enzymes and reductants under the environment with a pH value of about 5.0 40-42. To examine the cellular uptake and intracellular distribution of these as-prepared nanocomposites, the chemical cross-link of CaO_2_@HMSNs-PAA and FITC was performed to construct CaO_2_@HMSNs-PAA-FITC, which emitted green fluorescence upon excitation with a laser at 494 nm 41, 48. After PC-3 cells were incubated with CaO_2_@HMSNs-PAA-FITC for 0.5 h, 1 h and 3 h, the lysosomes were labeled by LysoTracker Red, which is lysosome-specific and exhibits red fluorescence when excited by a laser at 577 nm. Then, the cells were fixed, and the nuclei were stained with DAPI, emitting blue fluorescence upon laser excitation at 358 nm. The intracellular distribution of CaO_2_@HMSNs-PAA in PC-3 cells after endocytosis was observed CLSM 41. As shown in Figure 3, FITC-labeled CaO_2_@HMSNs-PAA could be visualized as green fluorescent spots inside of the PC-3 cells, and the results of Pearson's correlation analysis showed that there was a significant correlation between the uptake of CaO_2_@HMSNs-PAA by PC-3 cells and the time of incubation (*r =* 0.9972, *p <*0.05). Moreover, the green fluorescence from the CaO_2_@HMSNs-PAA-FITC overlapped with the red fluorescence of LysoTracker from the lysosomes surrounding the blue nuclei stained by DAPI, indicating that the as-prepared nanocomposites were internalized into PC-3 cells and localized in the much more acidic lysosomes where HMSNs would be gradually degraded 29, 65 and more H_2_O_2_ derived from the dual pH-responsive CaO_2_@HMSNs-PAA would form ROS of another kind. Furthermore, all of these intracellular ROS would be speculated to induce significant oxidative stress-related apoptosis due to the relatively weak ROS scavenging activity of tumor cells with a higher basal level of ROS.

### *In vitro* cytotoxicity of CaO_2_@HMSNs-PAA

**Figure 4:**
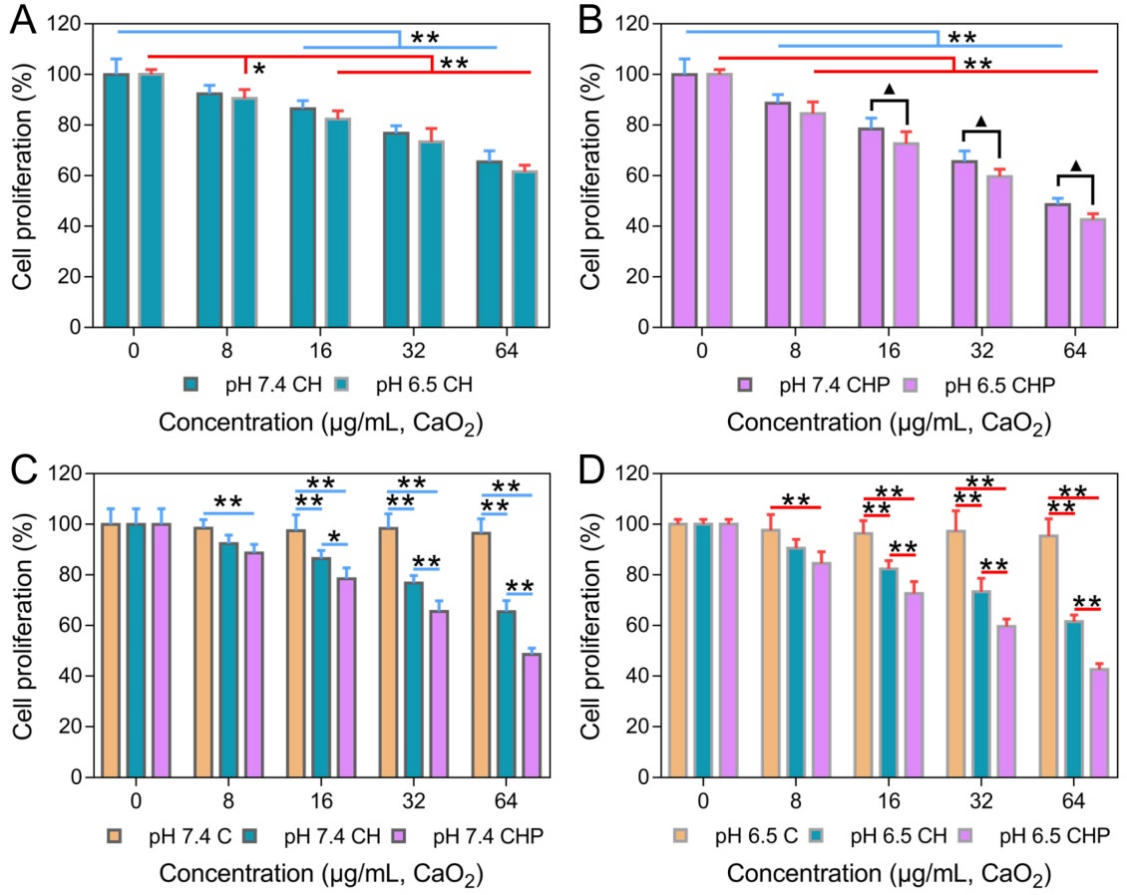
***In vitro* cytotoxicities of CaO_2_@HMSNs and CaO_2_@HMSNs-PAA against PC-3 cells.** (**A-B**) *In vitro* cytotoxicities of CaO_2_@HMSNs (A) and CaO_2_@HMSNs-PAA (B) against PC-3 cells in F-12K medium with pH values of 7.4 and 6.5 (*, *p <* 0.05, *vs.* the 0 µg/mL group; **, *p <* 0.01, *vs.* the 0 µg/mL group; ▲, *p <* 0.05, comparison between the groups treated with the same nanocomposite at different pH values). (**C-D**) Comparison of the *in vitro* cytotoxicities of CaO_2_, CaO_2_@HMSNs and CaO_2_@HMSNs-PAA dispersed in F-12K medium with pH values of 7.4 (C) and 6.5 (D) against PC-3 cells (*, *p <* 0.05; **, *p <* 0.01). Data are presented as the mean ± SD (n = 5).

The *in vitro* cytotoxicities of HMSNs, HMSNs-PAA, CaO_2_, CaO_2_@HMSNs and CaO_2_@HMSNs-PAA were evaluated by the CCK-8 proliferation assay. As shown in Figure S9A-B, both HMSNs and HMSNs-PAA exhibited no obvious cytotoxicity against PC-3 cells, even when the concentrations reached 200 μg/mL after 24 h of incubation (*p >* 0.05). These results demonstrated that both HMSNs and HMSNs-PAA exhibited outstanding biocompatibility and indicated the excellent availability of the two nanoparticles as ideal carriers for drug loading, which coincided with previously reported results 36, 48. While the cytotoxicities of CaO_2_, CaO_2_@HMSNs and CaO_2_@HMSNs-PAA enhanced as the material concentration increased to different degrees, although the increasing trend of cytotoxicity in the CaO_2_ group was nearly negligible (Figure S9C and Figure 4). In addition, both CaO_2_@HMSNs and CaO_2_@HMSNs-PAA with the equivalent CaO_2_ concentration of 64 μg/mL exhibited much higher cytotoxicity than CaO_2_. After 24 h of coculture with CaO_2_@HMSNs and CaO_2_@HMSNs-PAA in F-12K media (pH 7.4), the proliferation of PC-3 cells reduced to 65.50% and 48.50%, respectively (*p <* 0.01, *vs.* the 0 μg/mL group). However, cell proliferation remained at 96.48% when exposed to CaO_2_. Figure 4A-B shows similar results at equivalent CaO_2_ concentrations of 8 μg/mL, 16 μg/mL and 32 μg/mL.

The above cytotoxicity differences induced by the H_2_O_2_ from the free CaO_2_ and the loaded CaO_2_ could be partly ascribed to the different ultimately intracellular ROS levels after incubation with the two ROS sources. The free CaO_2_ would react with H_2_O after exposure to the culture medium, so most of the free CaO_2_ would be decomposed before entering into the cells. The extracellular H_2_O_2_ produced by the free CaO_2_ can be internalized into cells through passive diffusion 36, but the amount of H_2_O_2_ that could pass cell membranes is still limited by cells. During the diffusion, part of the H_2_O_2_ would be lost *via* decomposition before internalization into the cells, resulting in only a small amount of cellular damage. However, once encapsulated in the nanocarriers, the loading by HMSNs and coating by PAA could isolate the loaded CaO_2_ from H_2_O to varying degrees. Due to the encapsulation, the loaded CaO_2_ can enter the cells *via* nonspecific fluid-phase pinocytosis or adsorptive endocytosis 38. Furthermore, according to the published literature, silica nanoparticles have a high affinity for the hydrophilic head polar of phospholipids on the cell membrane 66. The loss before cellular uptake could be minimized by encapsulation, so CaO_2_ loaded in the above nanocomposites could concentratedly generate more H_2_O_2_ in the intracellular environment. All of these ROS would cause significant cytotoxicity by producing severe damage to proteins, DNA, lipids and other biomolecules, which would lead to much higher cytotoxicity than the free CaO_2_.

Moreover, the pH-responsive CaO_2_ release profile of CaO_2_@HMSNs-PAA is presumed to result in the TME-dependent cytotoxicity. To further confirm the pH-related tumor cytotoxicity, PC-3 cells were incubated with CaO_2_@HMSNs-PAA dispersed in F-12K medium with a pH of 6.5 to simulate the TME 50. As shown in Figure 4B, after exposure to CaO_2_@HMSNs-PAA with an equivalent CaO_2_ concentration of 8 μg/mL for 24 h, the cell proliferation would reduce from 88.68% to 84.59% if the pH value of culture medium was adjusted from 7.4 to 6.5. Besides, more obvious changes in cell proliferation, from 78.50% to 72.60%, from 65.57% to 59.61% and from 48.50% to 42.61%, were observed when the pH value of the F-12K medium decreased from 7.4 to 6.5 at equivalent CaO_2_ concentrations of 16 μg/mL, 32 μg/mL and 64 μg/mL, respectively (*p <* 0.05). Based on the above results, CaO_2_@HMSNs-PAA displayed excellent TME-dependent cytotoxicity. For comparison, the cytotoxicities of CaO_2_ and CaO_2_@HMSNs were also evaluated in F-12K medium with different pH values. Compared with CaO_2_@HMSNs-PAA, the cytotoxicities of both CaO_2_ and CaO_2_@HMSNs exhibited limited pH-responsiveness and no significant toxicity difference in F-12K medium with pH values of 7.4 and 6.5 (*p >* 0.05) (Figure S9C and Figure 4A), which was attributed to the severe loss before cellular internalization despite the pH-responsive release of H_2_O_2_ from CaO_2_. Moreover, Figure S9A-B demonstrates that naked nanocarriers, including HMSNs and HMSNs-PAA, showed no pH-responsive cytotoxicity (*p >* 0.05).

As mentioned above, tumor cells are more sensitive to the increase of exogenous ROS and are more prone to suffer oxidative stress. On the contrary, normal cells are significantly more resistant to the increased exogenous ROS than tumor cells due to the effective and efficient antioxidant system. According to Figure S10, all the above materials had no significant cytotoxicity against RWPE-1 cells at pH 7.4, suggesting that CaO_2_@HMSNs-PAA has excellent biosafety in normal cells under the simulated physiological environment (*p >* 0.05). Figure S11 illustrates that, after the supplement of catalase, the cytotoxicity of CaO_2_@HMSNs-PAA against PC-3 cells almost disappeared (*p <* 0.01), which indicated that the Ca^2+^ derived from CaO_2_@HMSNs-PAA could not cause obvious cytotoxicity. So, the antitumor effect of CaO_2_@HMSNs-PAA is mainly based on ROS rather than Ca^2+^.

All of the above results showed that, compared with the free CaO_2_ without obvious biotoxicity at low concentrations, CaO_2_@HMSNs-PAA exhibited distinct cytotoxicity under different pH conditions, which could be comprehensively attributed to the cellular uptake pattern, pH-responsive CaO_2_ release profiles and pH-responsive H_2_O_2_ generation from CaO_2_, leading to excellent TME-responsive cytotoxicity and remarkable alleviation of damages to normal tissues. Finally, as the lowest concentration with significant pH-responsiveness among the serial dilutions, an equivalent CaO_2_ concentration of 16 μg/mL was set as the working concentration in the subsequent *in vitro* assays.

### Exploration of the mechanism of CaO_2_@HMS-PAA-induced cytotoxicity

#### Elevating intracellular ROS

**Figure 5:**
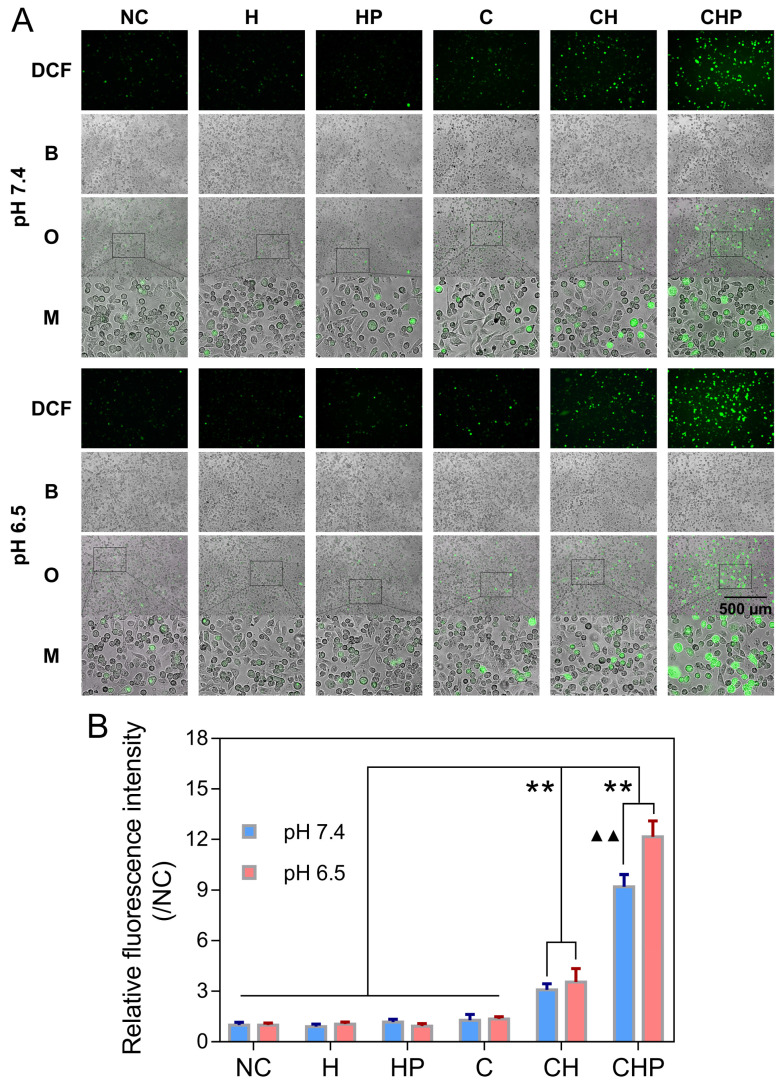
** Intracellular ROS generation in PC-3 cells after different treatments.** (**A**) Fluorescence microscopy images of PC-3 cells demonstrating the intracellular ROS generation (green fluorescence) after incubation with HMSNs (H), HMSNs-PAA (HP), CaO_2_ (C), CaO_2_@HMSNs (CH) and CaO_2_@HMSNs-PAA (CHP) dispersed in F-12K medium at pH 7.4 and 6.5 (NC, negative control; DCF, 2',7'-dichlorofluorescein; B, bright field; O, overlay; M, local magnified view of the overlay image). Scale bar, 500 µm. (**B**) Relative fluorescence intensities of DCF in PC-3 cells after incubation with H, HP, C, CH and CHP under the conditions of pH 7.4 and 6.5 (**, *p <* 0.01, *vs.* the groups of NC, H, HP and C; ▲▲, *p <* 0.01, comparison between the groups treated with the same nanocomposite at different pH values). Data are presented as the mean ± SD (n = 3).

The oxidatively sensitive nonfluorescent probe DCFH-DA is widely utilized to measure intracellular ROS levels. DCFH-DA itself is nonfluorescent, but after entering cells, it will be transformed to 2',7'-dichlorodihydrofluorescein (DCFH), which can be oxidized by intracellular ROS to generate DCF with a stable and high fluorescence, indicating the overall intracellular ROS levels 36, 63. A ROS assay kit based on DCFH-DA and the fluorescence microscopy were utilized to further clarify that the cytotoxicity of CaO_2_@HMSNs-PAA attributed to the ROS generated from the loaded CaO_2_. As shown in Figure 5A, compared with the negligible green fluorescence due to autoxidation in the negative control group, the cells treated with the free CaO_2_ or CaO_2_-loaded nanoparticles showed DCF fluorescence of different intensities, among which CaO_2_@HMSNs-PAA contributed the highest levels of fluorescence, although only extremely weak fluorescence could be observed when the cells were exposed to the free CaO_2_. After the pH value of the exposure medium was adjusted from 7.4 to 6.5 to simulate the TME, the fluorescence images exhibited more noticeable fluorescence intensity after the incubation with CaO_2_@HMSNs-PAA, while both the free CaO_2_ and CaO_2_@HMSNs treatment displayed a quite limited pH-responsiveness. To further prove that the noticeable intracellular fluorescence was derived from the CaO_2_ loaded into the as-prepared nanocomposites instead of the nanocarriers themselves, PC-3 cells were incubated with HMSNs or HMSNs-PAA *via* a similar process. However, no additional obvious intracellular fluorescence could be detected after exposure to the above nanocarriers by comparison with the negative control group. Therefore, after exposure to CaO_2_@HMSNs-PAA, the observed green fluorescence was attributed to the intracellular ROS from the loaded CaO_2_ rather than HMSNs or PAA. According to Figure 5B, CaO_2_@HMSNs-PAA could significantly elevate the intracellular level of ROS in PC-3 cells with significant pH-responsiveness (*p <* 0.01). All the above results were consistent with the evaluations of the *in vitro* cytotoxicity, so it could be reasonably speculated that the high level of intracellular ROS that could not be effectively eliminated by the overburdened antioxidant system in tumor cells would cause severe oxidative stress resulting in cytotoxicity *via* the induction of apoptosis.

#### Inducing the apoptosis of tumor cells

**Figure 6:**
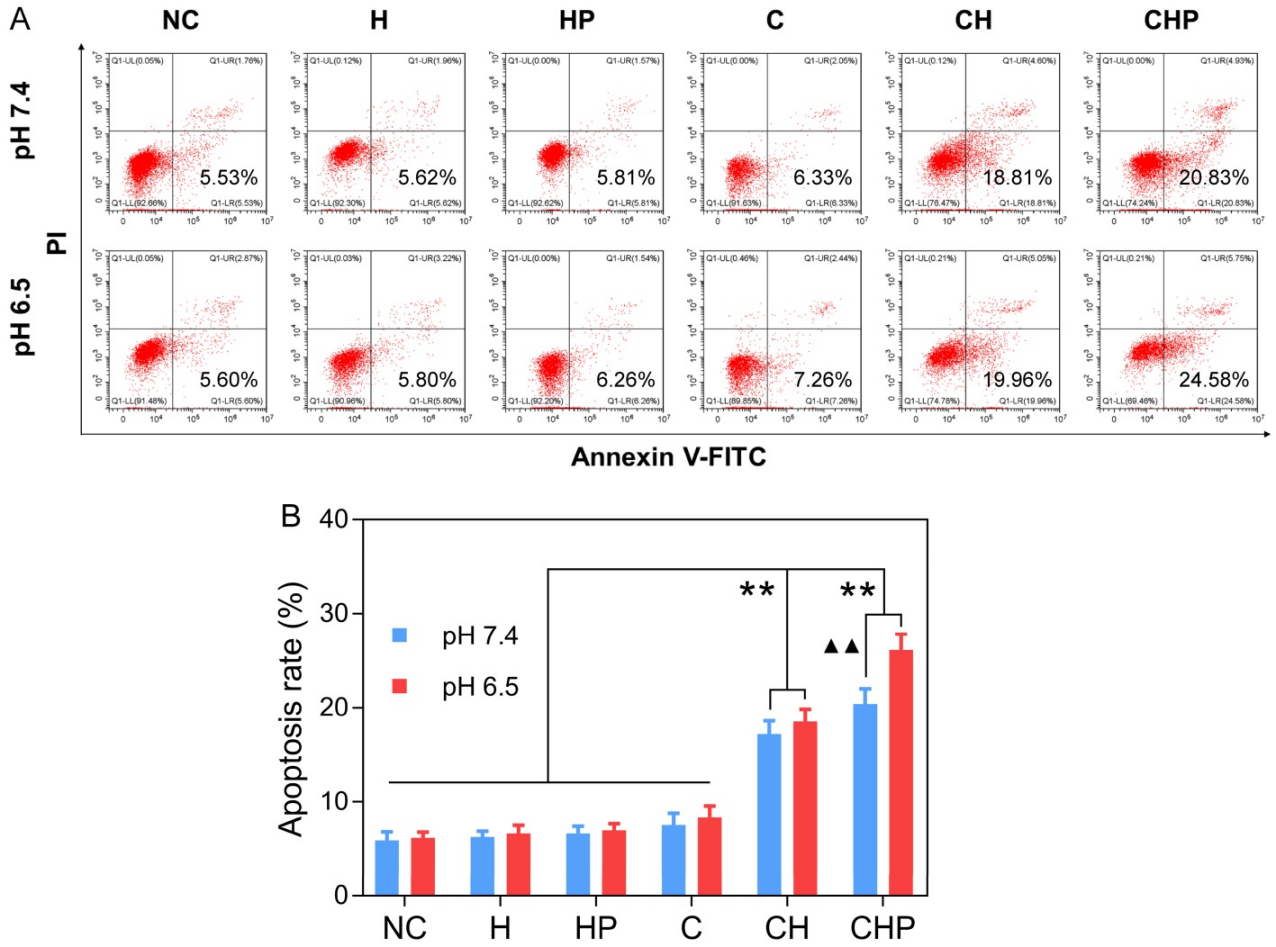
** Apoptosis of PC-3 cells after different treatments.** (**A**) Flow cytometry (FCM) results of the apoptosis of PC-3 cells after incubation with HMSNs (H), HMSNs-PAA (HP), CaO_2_ (C), CaO_2_@HMSNs (CH) and CaO_2_@HMSNs-PAA (CHP) dispersed in F-12K medium at pH 7.4 and 6.5 (NC, negative control). (**B**) Statistical analysis of the apoptosis rates of PC-3 cells after incubation with H, HP, C, CH and CHP under the conditions of pH 7.4 and 6.5 (**, *p <* 0.01, *vs.* the groups of NC, H, HP and C; ▲▲, *p <* 0.01, comparison between the groups treated with the same nanocomposite at different pH values). Data are presented as the mean ± SD (n = 3).

FCM based on Annexin-V-FITC/PI staining was adopted to evaluate the cell apoptosis induced by CaO_2_@HMSNs-PAA. Apoptotic cells were considered to be those situated in the lower right quadrants 67. As shown in Figure 6, under the condition of pH 7.4, the gourps of CaO_2_@HMSNs-PAA and CaO_2_@HMSNs exhibited significantly higher apoptosis rates of 20.42% and 17.22% than that of only 7.52% in the CaO_2_ group (*p <* 0.01). Furthermore, when the pH value of the F-12K medium decreased from 7.4 to 6.5, both the CaO_2_ group and the CaO_2_@HMSNs group showed only limited changes in the apoptosis rate, which rose slightly to 8.34% and 18.55%, respectively. However, more cells in the CaO_2_@HMSNs-PAA group were detected in the lower right quadrant (26.11% at pH 6.5 *vs.* 20.42% at pH 7.5, *p <* 0.01), which indicated the notable pH-responsiveness of CaO_2_@HMSNs-PAA in inducing the apoptosis of PC-3 cells. Moreover, no obvious apoptosis-inducing effect could be observed when the PC-3 cells were exposed to HMSNs or HMSNS-PAA dispersed in F-12K medium at a pH value of 7.4 or 6.5 (*p >* 0.05, *vs.* the negative control group). Thus, in accordance with the above results of both the cytotoxicity and ROS generation, it was clearly concluded that the designed CaO_2_@HMSNs-PAA could efficiently exhibit prominent TME-responsive cytotoxicity by inducing apoptosis *via* oxidative stress derived from the loaded CaO_2_.

#### Activating the mitochondrial apoptosis pathway

**Figure 7:**
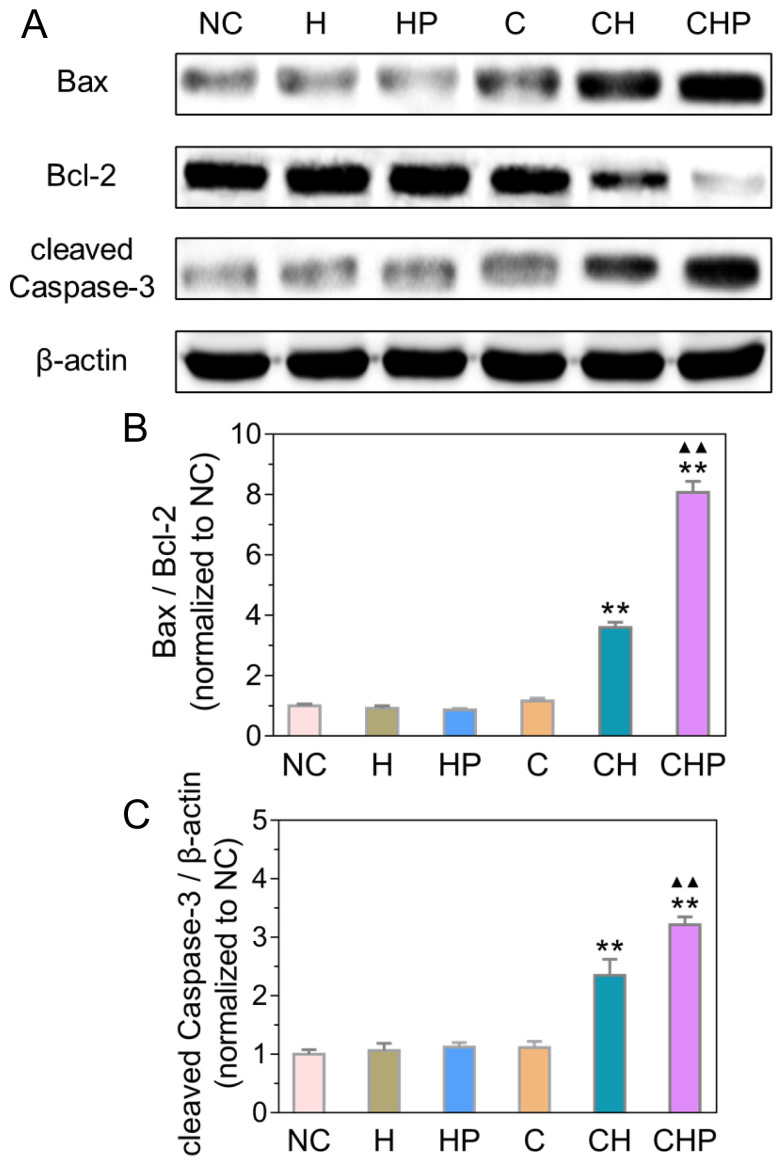
** Expressions of Bax, Bcl-2 and cleaved Caspase-3 in PC-3 cells after different treatments under the simulated tumor microenvironment.** (**A**) Western blot analysis of expressions of Bax, Bcl-2 and cleaved Caspase-3 in PC-3 cells treated with HMSNs (H), HMSNs-PAA (HP), CaO_2_ (C), CaO_2_@HMSNs (CH) and CaO_2_@HMSNs-PAA (CHP). The β-actin served as an internal control. NC, negative control. (**B-C**) Expression ratio of Bax to Bcl-2 (B) and relative expression of cleaved Caspase-3 (C) in PC-3 cells treated with H, HP, C, CH and CHP (**, *p <* 0.01, *vs.* the groups of NC, H, HP and C; ▲▲, *p <* 0.01, *vs.* the CH group). Data are presented as the mean ± SD (n = 3).

Combined with the results of ROS generation and apoptosis in the above cellular assays, the observed satisfactory antitumor efficacy was speculated to derive from the apoptosis induced by oxidative stress *via* ROS. Oxidative stress can cause severe damage to the structure and function of cells through various approaches, among which the mitochondria-mediated apoptosis has been widely studied as one of the most important pathways 68, 69. Therefore, some critical molecules in the above pathway, including Bax, Bcl-2 and cleaved Caspase-3, were analyzed by Western blot analysis to explore the molecular mechanism of apoptosis induced by CaO_2_@HMSNs-PAA. Figure 7 illustrates that the ratios of Bax to Bcl-2 increased significantly in the two groups treated with CaO_2_-loaded nanoparticles (*p <* 0.01, *vs.* all the other groups), indicating the effective initialization of the mitochondria-mediated apoptosis. Similarly, compared with the negative control, HMSNs, HMSNs-PAA and CaO_2_, both CaO_2_@HMSNs and CaO_2_@HMSNs-PAA could significantly upregulate the expression of cleaved Caspase-3 (*p <* 0.01), which demonstrates the successful execution of the mitochondria-mediated apoptosis. These results coincided with the above evaluations of the intracellular ROS generation and the cellular apoptosis and further indicated that the mechanism of antitumor effects of CaO_2_@HMSNs-PAA was inducing mitochondria-mediated apoptosis *via* oxidative stress.

### *In vivo* biosafety of CaO_2_@HMSNs-PAA

The *in vivo* biosafety evaluation was performed on healthy BALB/c mice injected with intravenous CaO_2_@HMSNs-PAA at doses of 0 mg/kg, 10 mg/kg, 20 mg/kg and 40 mg/kg. The body weights recorded over the entire observation period of 30 days indicated that intravenous administration of CaO_2_@HMSNs-PAA caused no obvious influence on the mouse growth (Figure S12) (*p >* 0.05). Furthermore, both the liver function (ALT, AST and ALP) and the renal function (BUN and CREA) showed no significant differences between the groups at the various doses of the as-prepared nanoparticles on day 0, day 1, day 7 and day 30 (*p >* 0.05) (Figure S13), which revealed that CaO_2_@HMSNs-PAA had an inconspicuous impact on the liver and renal functions of the mice. Similarly, no observable change in WBC, RBC, HGB, HCT, MCV, MCH and MCHC could be detected at the time given above (*p >* 0.05) (Figure S13), elucidating that all the mice in the different groups had the same hematopoietic and immune status during the whole evaluation period. Moreover, H&E staining of the histological sections of the major organs (the heart, liver, spleen, lung and kidney) from all the groups at the indicated time showed no distinct pathological abnormalities (Figures S14-S17). According to the above comprehensive *in vivo* evaluations, CaO_2_@HMSNs-PAA exhibited excellent biocompatibility, attributable to the negligible influence on normal tissues by effectively eliminating the ROS derived from the loaded CaO_2_ *via* the complete antioxidant system in normal cells, which would ensure promising *in vivo* therapeutic applications. In addition, as the highest safe dose utilized in the biosafety evaluation, the equivalent CaO_2_@HMSNs-PAA dose of 40 mg/kg was regarded as the working dose in the *in vivo* antitumor therapeutic assays.

### Biodistribution of CaO_2_@HMSNs-PAA after intravenous administration

**Figure 8:**
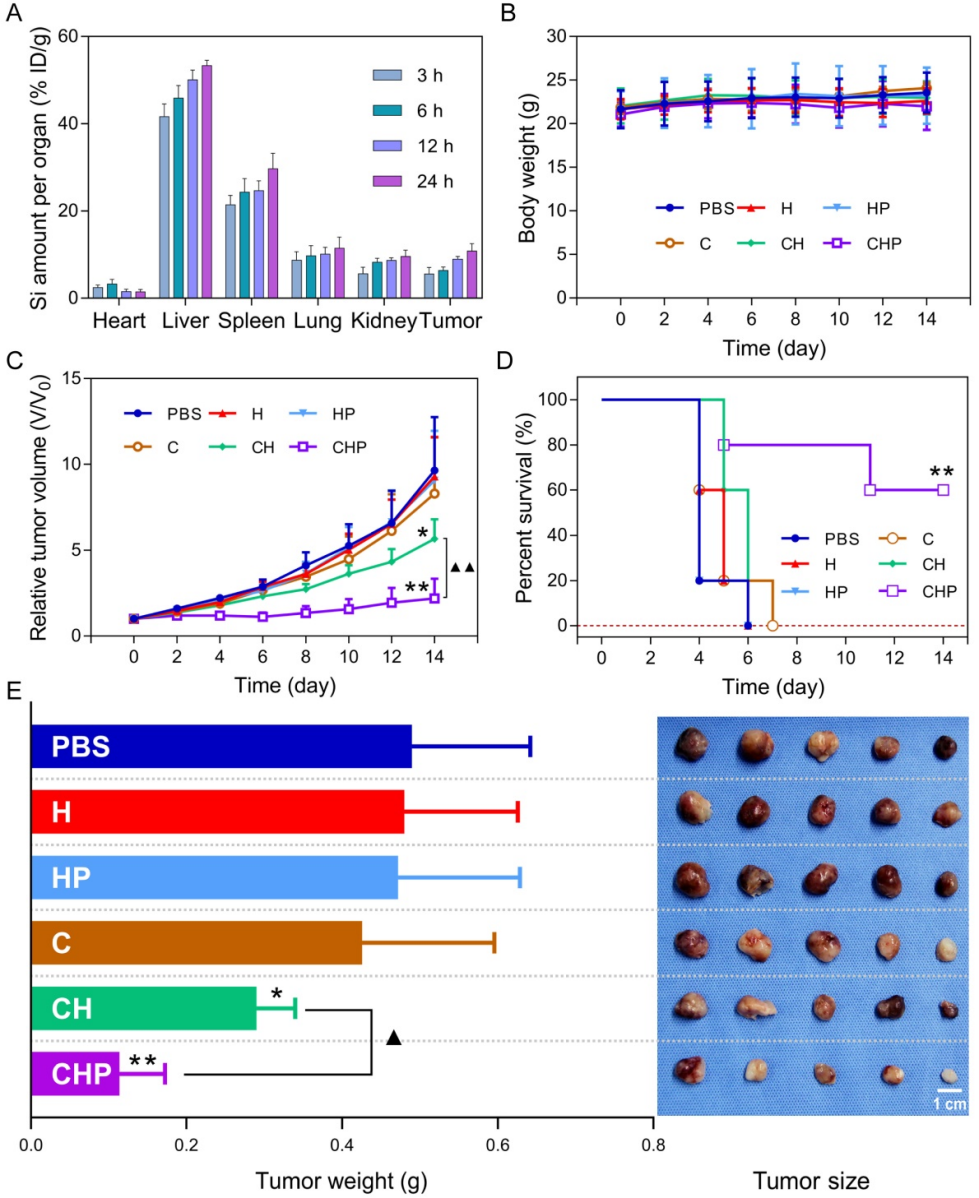
** Biodistribution and *in vivo* antitumor therapeutics of CaO_2_@HMSNs-PAA.** (**A**) Biodistribution of CaO_2_@HMSNs-PAA (CHP) in the subcutaneous PC-3 xenografted tumor-bearing BALB/c nude mice at 3 h, 6 h, 12 h and 24 h after intravenous injection (n = 3). (**B-C**) Body weights (B) and relative tumor volumes (C) of the PC-3 tumor-bearing nude mice recorded after intravenous administration of phosphate-buffered saline (PBS), HMSNs (H), HMSNs-PAA (HP), CaO_2_ (C), CaO_2_@HMSNs (CH) and CaO_2_@HMSNs-PAA (CHP) (n = 5) (*, *p <* 0.05, *vs.* the PBS group; **, *p <* 0.01, *vs.* the PBS group; ▲▲, *p <* 0.01, comparison between the CH group and the CHP group). (**D**) Kaplan-Meier survival analysis of the PC-3 tumor-bearing BALB/c nude mice, with the definition of survival as the tumor volume (V) failed to exceed two times of the initial volume (V_0_) (**, *p <* 0.01, *vs.* the PBS group). (**E**) Tumor weights and tumor sizes on day 14 after intravenous administration of PBS, H, HP, C, CH and CHP (n = 5) (*, *p <* 0.05, *vs.* the PBS group; **, *p <* 0.01, *vs.* the PBS group; ▲, *p <* 0.05, comparison between the CH group and the CHP group). Scale bar, 1 cm. Data are presented as the mean ± SD.

The biodistribution of CaO_2_@HMSNs-PAA in major organs and tumors of subcutaneous PC-3 xenografted tumor-bearing BALB/c nude mice was detected based on ICP-MS at 3 h, 6 h, 12 h and 24 h after injection *via* the tail vein 23, 57, 63. From Figure 8A, intravenously injected CaO_2_@HMSNs-PAA were mainly distributed in the liver and spleen due to capture by the reticuloendothelial system 63. Simultaneously, the accumulation of this nanoparticle in the tumors was initially 5.57% at 3 h, then 6.39% at 6 h and finally 10.84% at 24 h after intravenous administration because of the EPR effect 63.

### *In vivo* antitumor therapeutics of CaO_2_@HMSNs-PAA

The subcutaneous PC-3 xenografted tumor model was established before intravenous injection of different materials, according to which the PC-3 tumor-bearing BALB/c nude mice were randomly assigned into six groups including the PBS group (control), the HMSNs group, the HMSNs-PAA group, the CaO_2_ group, the CaO_2_@HMSNs group and the CaO_2_@HMSNs-PAA group. As shown in Figure 8C-D, after administration of the given material *via* the tail vein, significant inhibition of the tumor growth during the whole evaluation period of 14 days could be observed in both the CaO_2_@HMSNs-PAA group (*p <* 0.01, *vs.* the PBS group) and the CaO_2_@HMSNs group (*p <* 0.05, *vs.* the PBS group), and CaO_2_@HMSNs-PAA displayed more remarkable tumor inhibition efficacy than CaO_2_@HMSNs (*p <* 0.01). In contrast, no obvious influence on tumor volume was observed in the HMSN group, the HMSN-PAA group and the CaO_2_ group. Similar results were also obtained upon the evaluation of inhibition of tumor weights and sizes on day 14 (Figure 8E), indicating effective inhibition of the tumor growth by intravenous administration of CaO_2_@HMSNs-PAA. All of these *in vivo* tumor inhibition results from the different treatments were consistent with the above evaluations of *in vitro* cytotoxicity. Moreover, the ignorable influence on the body weights of all the groups further proved the notable biocompatibility of the as-prepared nanoparticles (Figure 8B).

Afterwards, the tumors and major organs (the heart, liver, spleen, lung and kidney) of all the mice were extracted on day 14, followed by histological analysis to further confirm the tumor-suppressing effects of the nanosystem. As shown in Figure S18, compared with the PBS group, the hematoxylin and eosin (H&E) staining sections from the groups treated with the CaO_2_-loaded nanocomposites exhibited notable structural damage of the tumor tissue, especially the almost complete destruction in the CaO_2_@HMSNs-PAA group. However, no observable variation could be detected when the mice were treated with the free CaO_2_, HMSNS and HMSNs-PAA. Meanwhile, the major organs of the mice from all the groups displayed negligible histological changes. Based on the above evaluation of the *in vivo* antitumor therapeutics, CaO_2_@HMSNs-PAA exhibited prominent TME-responsive antitumor efficacy without detectable side injuries to normal tissues, which coincided with the above cellular assays and biosafety evaluation.

To further determine the *in vivo* apoptosis mechanism, the expression of Bax, Bcl-2 and cleaved Caspase-3 in tumor tissues were analyzed by immunohistochemistry. Because of the increased ROS generation in the early stage, all tumors of another eighteen PC-3 tumor-bearing BALB/c nude mice were obtained for immunohistochemical analysis of the above apoptosis markers on day 2 after the intravenous administration of the above materials. Figure S19 showed that, compared with the other groups, higher expressions of the apoptosis promotor Bax and the apoptosis executioner cleaved Caspase-3 accompanied by lower expression of the apoptosis inhibitor Bcl-2 could be observed in the CaO_2_@HMSNs-PAA group, which coincided with the results of the *in vitro* Western blot analysis. In addition, compared with the other groups, more apoptosis could be observed in the CaO_2_@HMSNs-PAA group according to the results of the TUNEL assay (Figure S19), which was consistent with the evaluation of apoptosis in PC-3 cells after incubation with these materials.

In summary, it is reasonable to conclude that CaO_2_@HMSNs-PAA could exert prominent TME-responsive *in vivo* antitumor therapeutics by inducing apoptosis *via* ROS initiating the mitochondria-mediated pathway, and this could minimize the damages to normal tissues because of the different ability in scavenging ROS. Although the injection of CaO_2_@HMSNs-PAA was conducted only once in this study, the promising results also provide a scientific basis for further improvement in the antitumor efficacy by multiple administrations or combination with other interventions in the future.

However, due to the limited availability of time, human resources, experimental conditions and equipment, this study still has many limitations. The orthotopic tumor xenograft model, which is more likely to mimic the growth environment for the tumor in human bodies, was not adopted to evaluate the *in vivo* anti-prostate cancer effect of CaO_2_@HMSNs-PAA. The evaluation of the safety and antitumor efficacy of CaO_2_@HMSNs-PAA *via* multiple administration was not conducted. The mechanism of CaO_2_@HMSNs-PAA against prostate cancer was not studied in detail. Although this study provides a promising concept for the treatment of prostate cancer, the efficacy of CaO_2_@HMSNs-PAA is not as ideal as some clinical antitumor drugs, such as docetaxel which has been widely used as one of the most potent antitumor therapy for prostate cancer with a significant clinical benefit in survival 70, 71. So, many efforts and breakthroughs are still needed to make in our subsequent researches.

## Conclusion

In this work, we developed an effective and biocompatible ROS-controlled release nanosystem with TME-responsiveness by loading CaO_2_ to PAA-coated HMSNs. The EPR effect allowed for the relative enrichment of CaO_2_@HMSNs-PAA in the tumor tissue, the pH-responsive reactivity of CaO_2_ itself and the pH-responsive switching of PAA contributed to the TME-responsive release of ROS. The above design ensured that ROS generation was more concentrated on tumor tissues and tumor cells, which could significantly reduce the loss during transportation and mitigate side injuries to normal tissues. *In vitro* assays revealed that CaO_2_@HMSNs-PAA could induce the apoptosis of PC-3 cells through the mitochondria-mediated pathway because of the efficiently responsive generation of ROS. Similarly, *in vivo* experiments demonstrated that the intravenous administration of CaO_2_@HMSNs-PAA could significantly inhibit the tumor growth in the subcutaneous PC-3 xenografted tumor model and activate the mitochondrial apoptosis pathway to induce apoptosis *via* ROS-initiating oxidative stress, simultaneously leading to no significant influences on the important blood indicators and major organ structures. In conclusion, based on the concept of “efficacy-shaping nanomedicine”, we provide a promising therapeutic strategy for the tumor-specific treatment of prostate cancer.

## Supplementary Material

Supplementary figures.

## Abbreviations

ALP: alkaline phosphatase
ALT: alanine aminotransferase
ANOVA: analysis of variance
APTES: 3-aminopropyl triethoxysilane
AST: aspartate aminotransferase
BET: Brunauer-Emmett-Teller
BJH: Barrett-Joyner-Halenda
BUN: blood urea nitrogen
CAT: catalase
CLSM: confocal laser scanning microscopy
CREA: creatinine
CTAB: cetyltrimethylammonium bromide
DAPI: 4,6-diamidino-2-phenylindole
DCF: 2',7'-dichlorofluorescein
DCFH: 2',7'-dichlorodihydrofluorescein
DCFH-DA: 2',7'-dichlorodihydrofluorescein diacetate
DLS: dynamic light scattering
ECL: enhanced chemiluminescence
EDS: energy-dispersive X-ray spectroscopy
EPR: enhanced permeability and retention
F-12K: Kaighn's Modification of Ham's F-12
FBS: fetal bovine serum
FCM: flow cytometry
FITC: fluorescein isothiocyanate
FT-IR: Fourier transform infrared
H&E: hematoxylin and eosin
HCT: hematocrit
HGB: hemoglobin
HMSNs: hollow mesoporous silica nanoparticles
HRP: horseradish peroxidase
ICP: inductively coupled plasma
ICP-MS: inductively coupled plasma-mass spectrometry
MCH: mean corpuscular hemoglobin
MCHC: mean corpuscular hemoglobin concentration
MCV: mean corpuscular volume
PAA: polyacrylic acid
PBS: phosphate-buffered saline
PCa: prostate cancer
PI: propidium iodide
PVDF: polyvinylidene difluoride
RIPA: radioimmunoprecipitation assay
RBC: red blood cells
ROS: reactive oxygen species
sSiO_2_: solid silica dioxide nanoparticles
SEM: scanning electron microscope
TBST: Tris-buffered saline/Tween-20
TEM: transmission electron microscopy
TEOS: tetraethyl orthosilicate
TME: tumor microenvironment
WBC: white blood cells
XRD: X-ray diffraction
