# Supplementary material for: Efficacy-shaping nanomedicine by loading Calcium Peroxide into Tumor Microenvironment-responsive Nanoparticles for the Antitumor Therapy of Prostate Cancer
Source: Theranostics. 2020 Aug 2;10(21):9808–29. doi: 10.7150/thno.43631 (PMC7449903; doi:10.7150/thno.43631)
Supplement: Supplementary file 1 — Supplementary figures. [file thnov10p9808s1.pdf]

Supporting Information for:

Efficacy-shaping nanomedicine by loading Calcium Peroxide into Tumor Microenvironment-responsive Nanoparticles for the Antitumor Therapy of Prostate Cancer

Di Wu [1,2 *], Zi-Qiang Zhu [1*], Hai-Xiao Tang [1], Zhi-En Shi [1], Jian Kang [1],

Qiang Liu [1 ✉], Jun Qi [1 ✉]

* These authors contributed equally to this work.

1. Department of Urology, Xinhua Hospital Affiliated to Shanghai Jiao Tong University School of Medicine, Shanghai 20092, China.

2. Department of Urology, Huadong Hospital Affiliated to Fudan University, Shanghai 20040, China.

✉ Correspondence:

Jun Qi, MD, Ph.D., Department of Urology, Xinhua Hospital Affiliated to Shanghai Jiao Tong University School of Medicine, Shanghai 20092, China, Tel: +86-21-2507-8090, Fax: +86-21-2507-8080, Email: qijun@xinhuamed.com.cn;

Qiang Liu, MD, Ph.D., Department of Urology, Xinhua Hospital Affiliated to Shanghai Jiao Tong University School of Medicine, Shanghai 20092, China, Tel: +86-21-2507-8090, Fax: +86-21-2507-8080, Email: lqws_sjtu@163.com.

Supplementary Figures (Figures S1-S19):

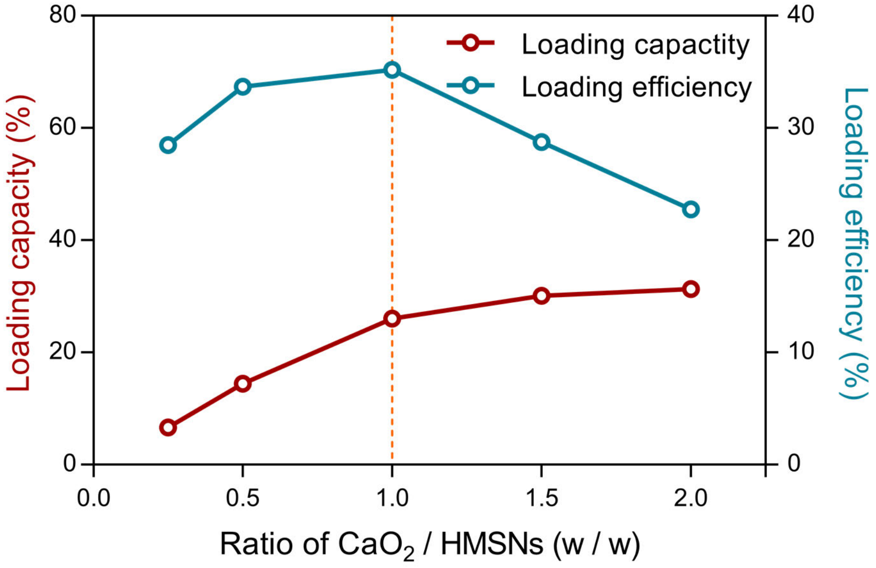

Figure S1. The influence of the mass ratio of CaO_2 to HMSNs on the loading capacity and the loading efficiency of CaO_2@HMSNs.

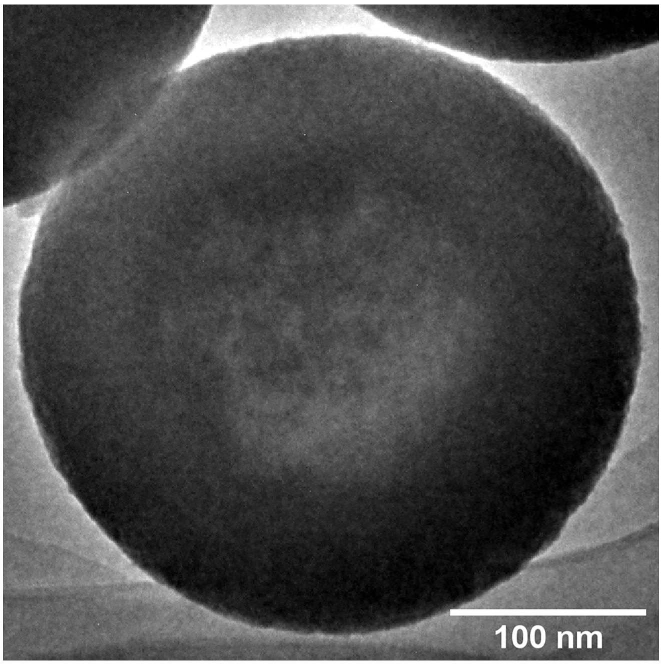

Figure S2. Transmission electron microscopy (TEM) image of CaO_2@HMSNs. Scale bar, 100 nm.

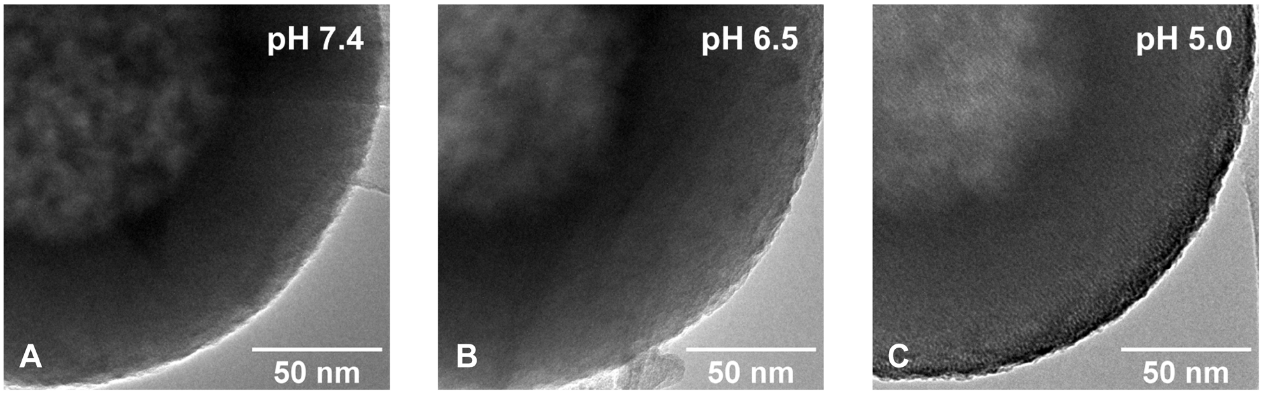

Figure S3. Transmission electron microscopy (TEM) images of CaO_2@HMSNs-PAA at different pH values. TEM images of CaO_2@HMSNs-PAA at pH values of 7.4 (**A**), 6.5 (**B**) and 5.0 (**C**). Scale bars, 50 nm.

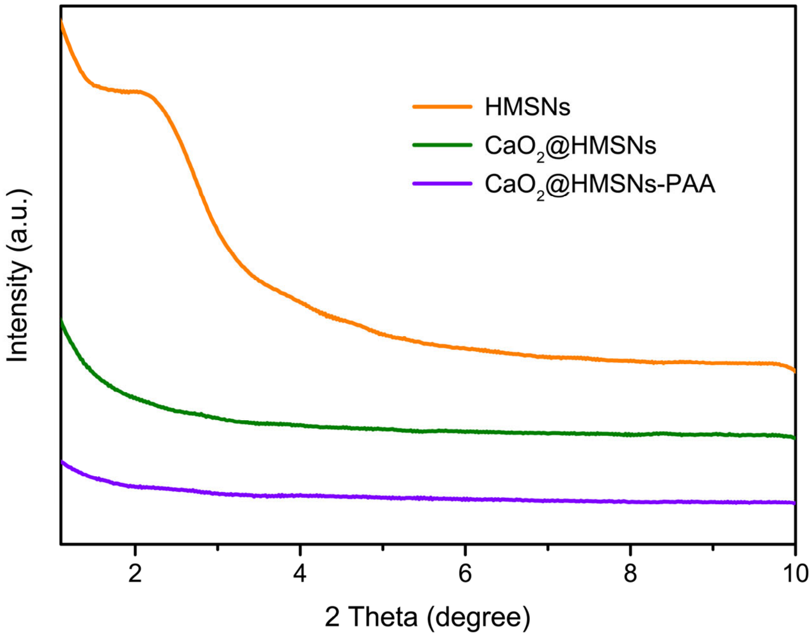

Figure S4. Low-angle X-ray diffraction (XRD) patterns of HMSNs, CaO_2@HMSNs and CaO_2@HMSNs-PAA.

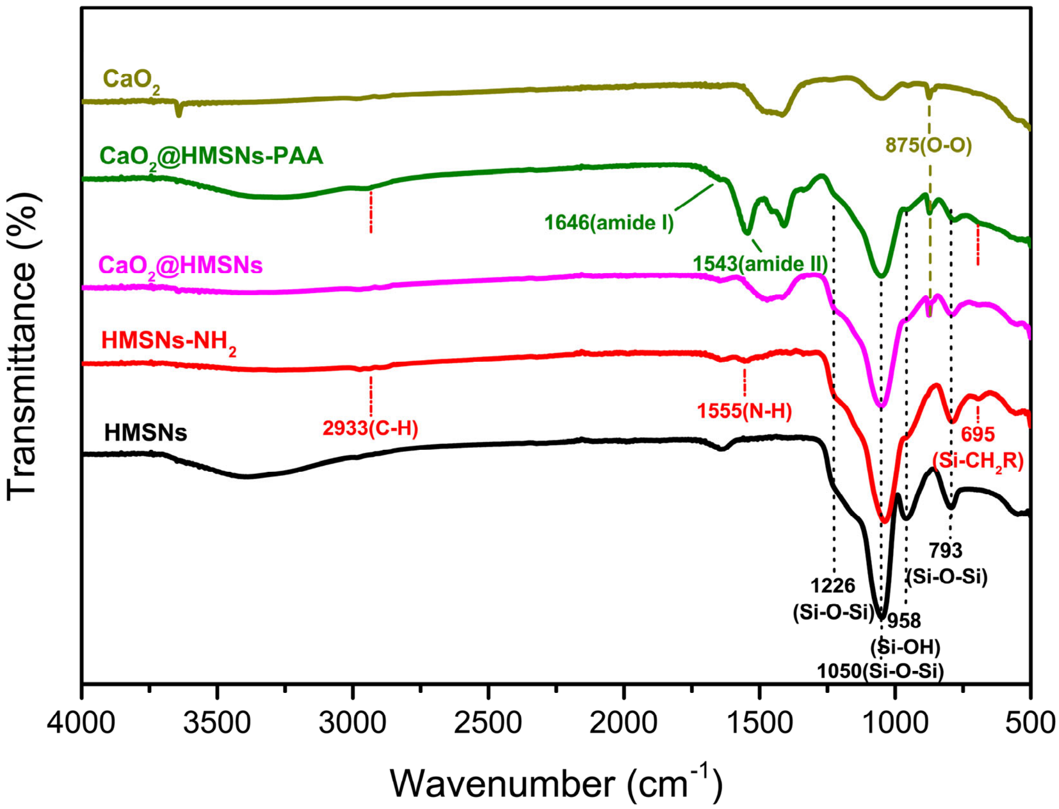

Figure S5. Fourier transform infrared (FT-IR) spectra of HMSNs, HMSNs-NH$_2$, CaO$_2$, CaO$_2$@HMSNs and CaO$_2$@HMSNs-PAA.

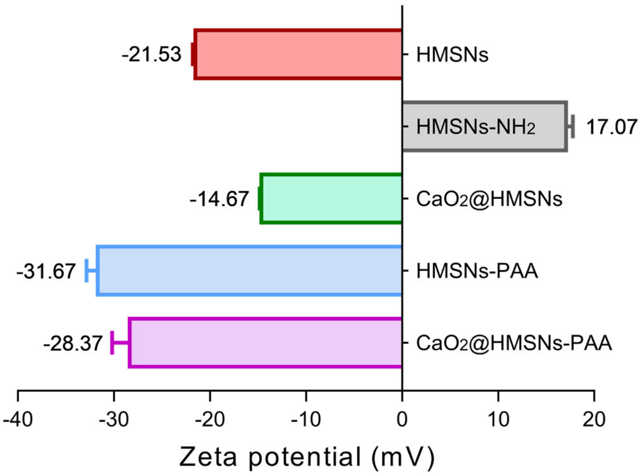

Figure S6. Zeta potentials of HMSNs, HMSNs-NH$_2$, CaO$_2$@HMSNs, HMSNs-PAA and CaO$_2$@HMSNs-PAA dispersed in ethanol.

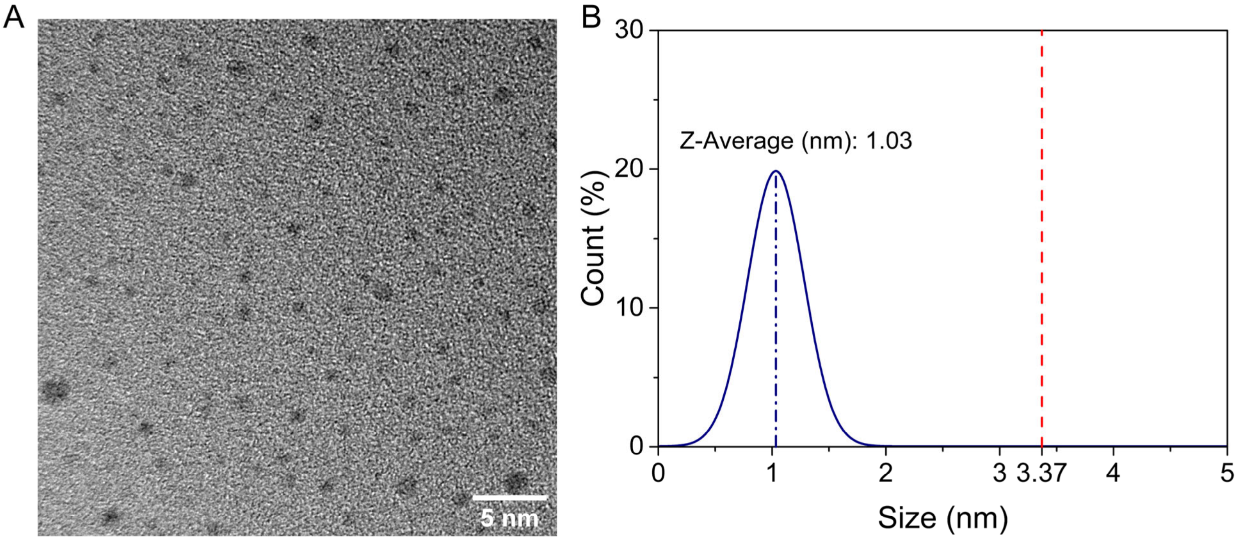

Figure S7. Transmission electron microscopy (TEM) image and particle size distribution of CaO_2 particles. (**A**) TEM image of CaO_2 particles. Scale bar, 5 nm. (**B**) Particle size distribution of CaO_2 particles. The red dash line indicates the average pore size of HMSNs (about 3.37 nm).

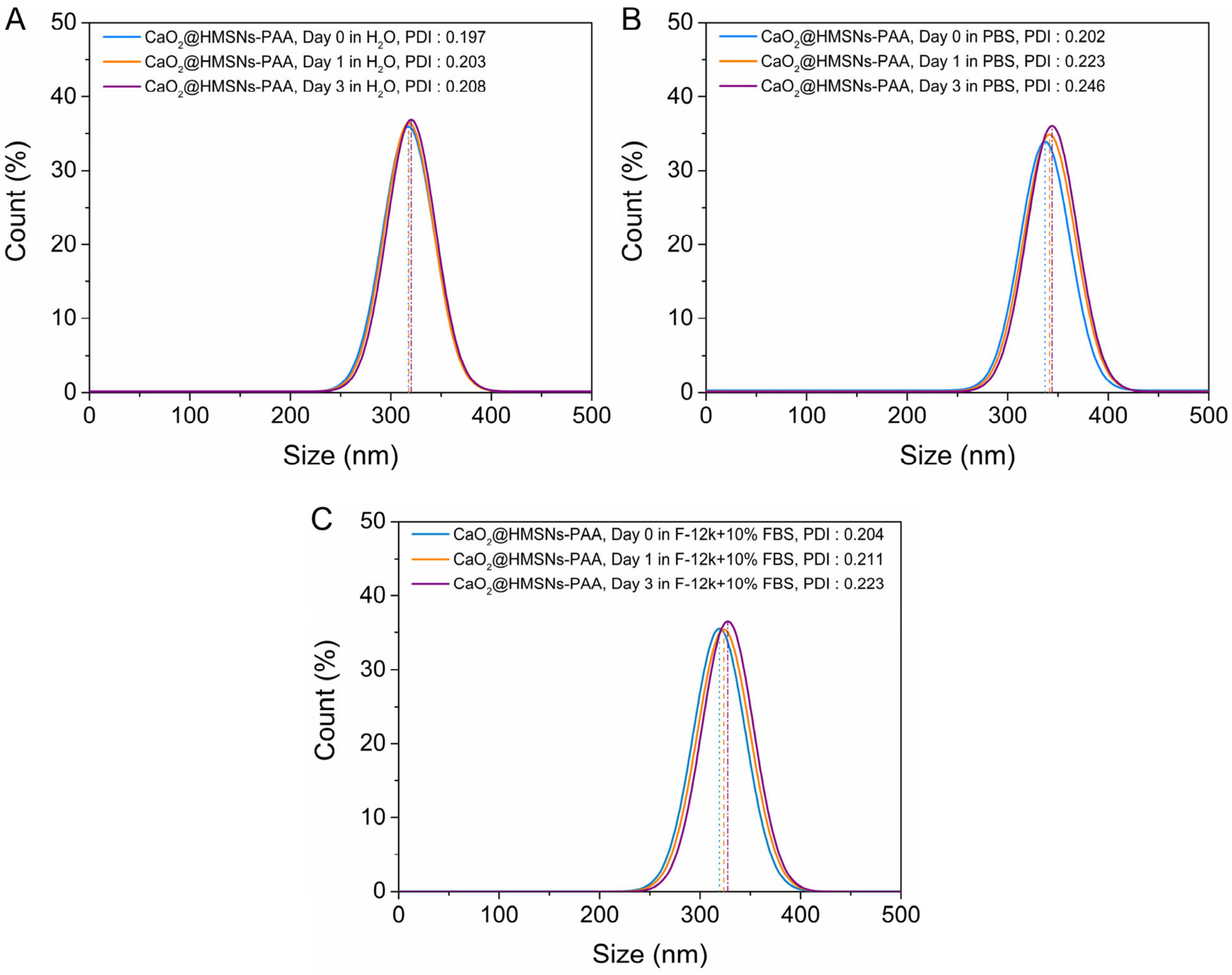

Figure S8. Dispersibility and stability of CaO_2@HMSNs-PAA in various buffers. Dynamic light scattering (DLS) particle size distributions and polydispersity indexes (PDI) of CaO_2@HMSNs-PAA in H_2O (**A**), phosphate-buffered saline (PBS) (**B**) and F-12K medium + 10% fetal bovine serum (FBS) (**C**) on day 0, day 1 and day 3.

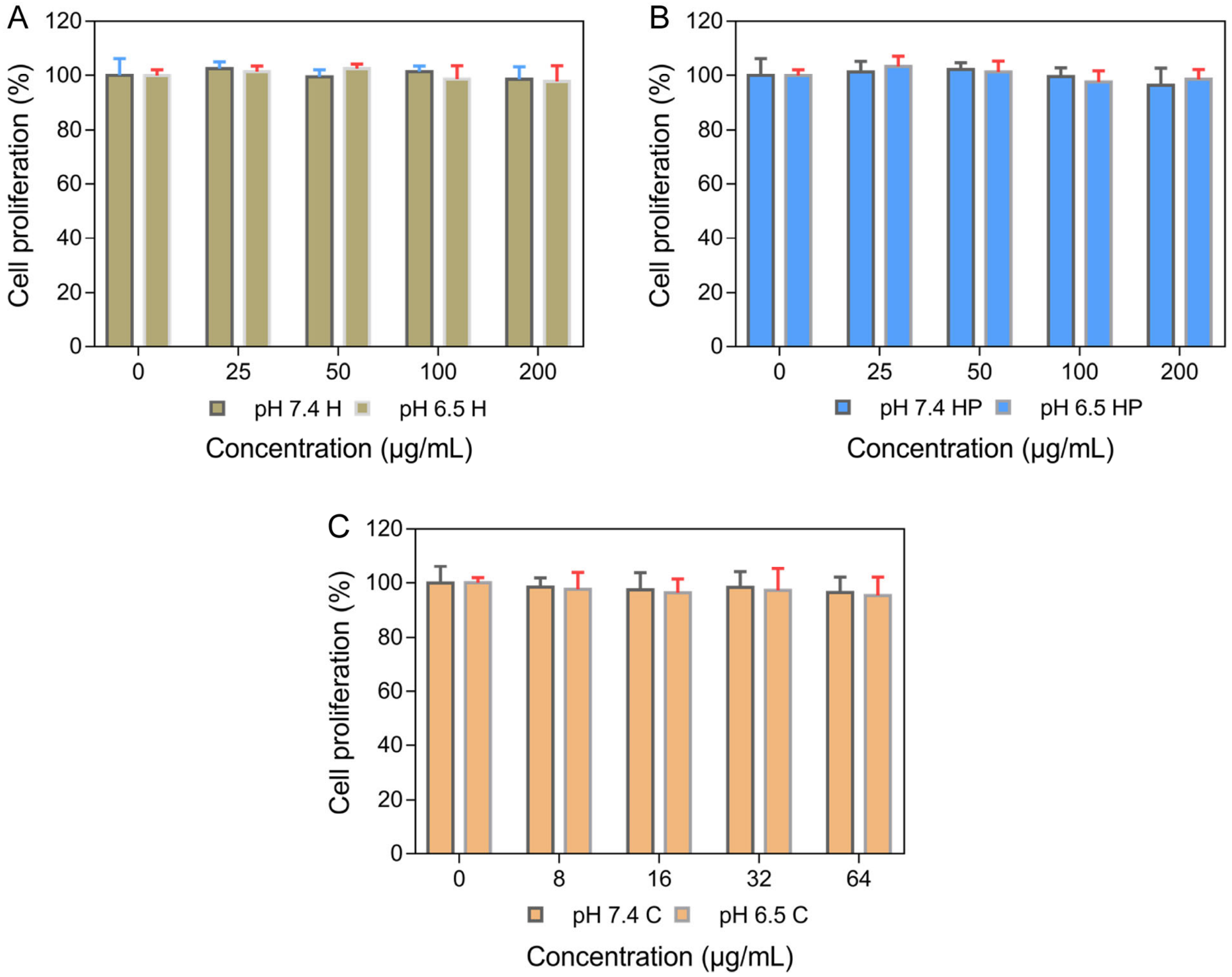

Figure S9. *In vitro* cytotoxicities of HMSNs, HMSNs-PAA and CaO_2 against PC-3 cells. Cell proliferation rates after incubation with HMSNs (**A**), HMSNs-PAA (**B**) and CaO_2 (**C**). Data are presented as the mean ± SD ($n = 5$, $p > 0.05$).

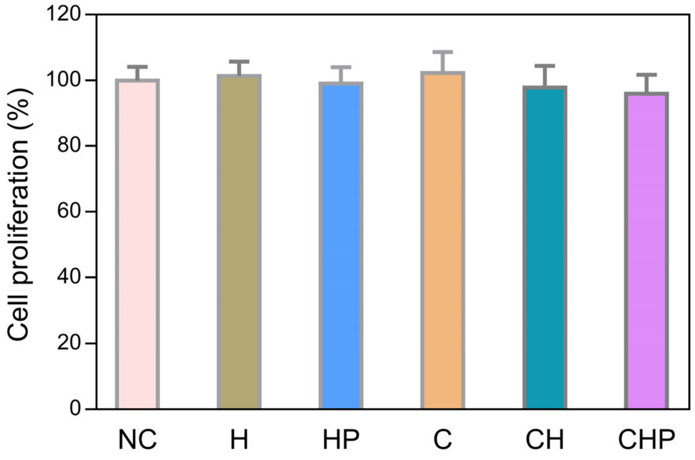

Figure S10. ***In vitro*** **cytotoxicities of HMSNs, HMSNs-PAA, CaO_2, CaO_2@HMSNs and CaO_2@HMSNs-PAA against RWPE-1 cells under the simulated physiological condition.** NC, negative control; H, HMSNs; HP, HMSNs-PAA; C, CaO_2; CH, CaO_2@HMSNs; CHP, CaO_2@HMSNs-PAA. Data are presented as the mean ± SD ($n = 5$, $p > 0.05$).

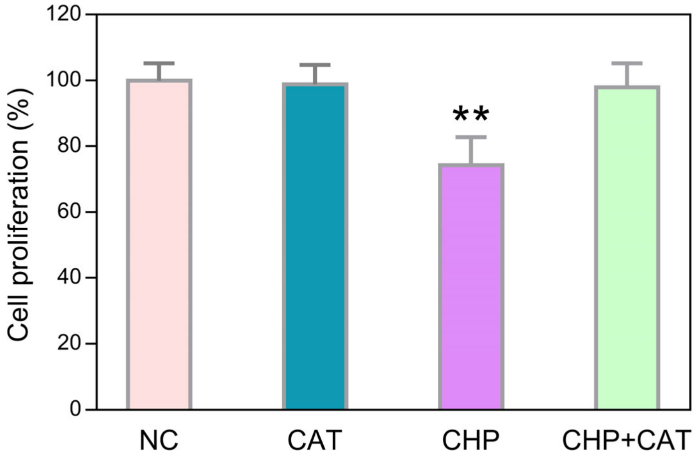

Figure S11. ***In vitro*** **cytotoxicity of CaO_2@HMSNs-PAA plus with catalase against PC-3 cells.** NC, negative control; CAT, catalase; CHP, CaO_2@HMSNs-PAA. Data are presented as the mean ± SD ($n = 5$; **, $p < 0.01$, *vs.* the NC group, the CAT group and the CHP+CAT group).

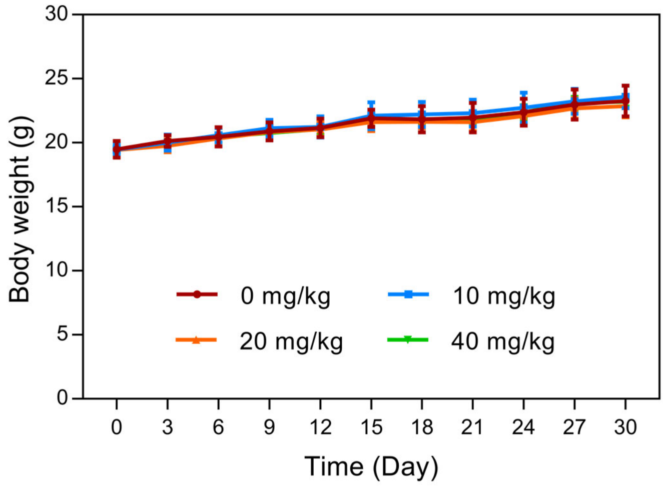

Figure S12. Body weights of BALB/c mice recorded during the measurement period of 30 days after intravenous administration of CaO_2@HMSNs-PAA with doses of 0 mg/kg, 10 mg/kg, 20 mg/kg and 40 mg/kg. Data are presented as the mean ± SD ($n = 5$, $p > 0.05$).

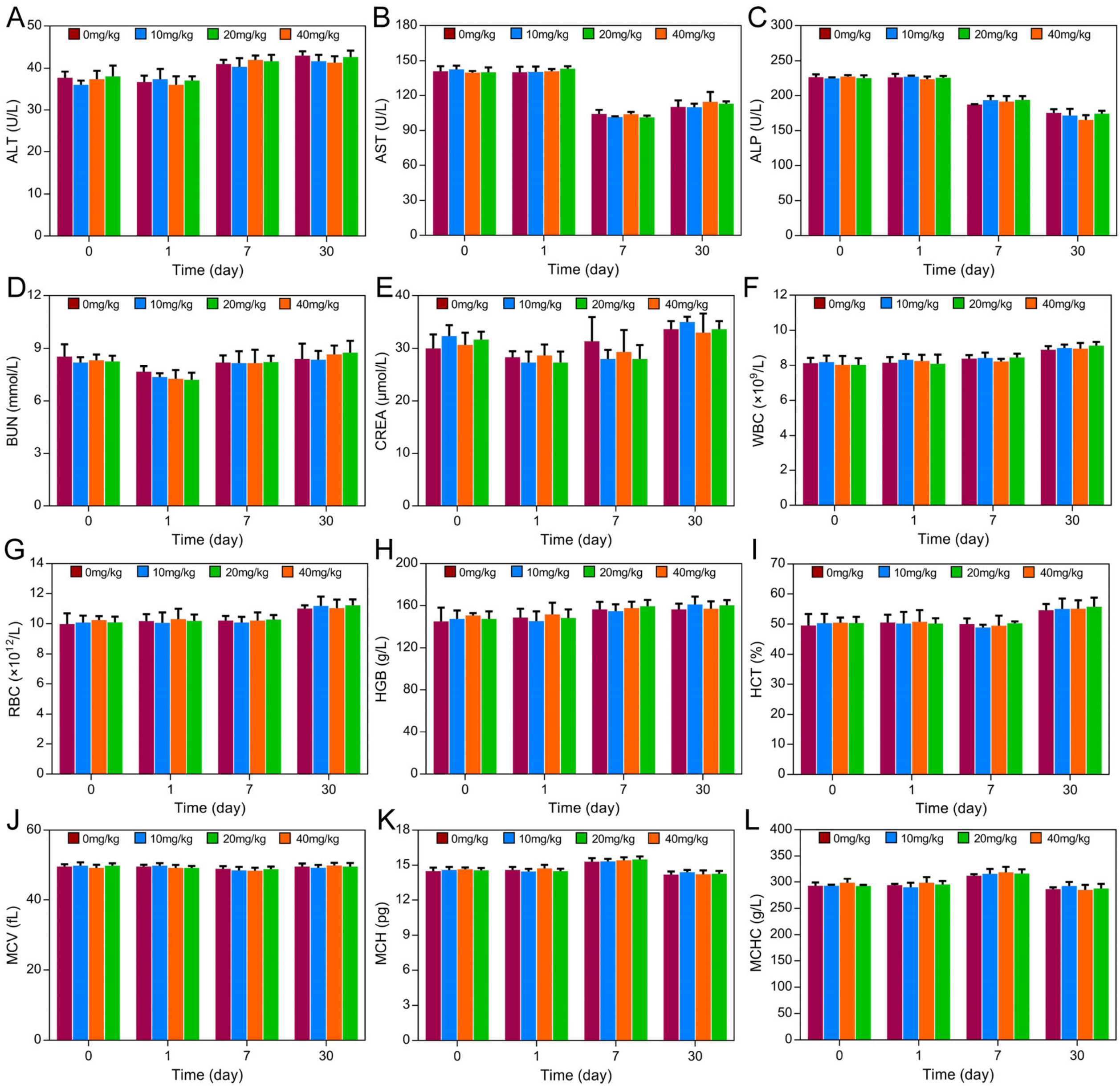

Figure S13. Liver function, renal function and blood panel parameters of BALB/c mice recorded on day 0, day 1, day 7 and day 30 after intravenous administration of CaO_2@HMSNs-PAA with doses of 0 mg/kg, 10 mg/kg, 20 mg/kg and 40 mg/kg. ALT (**A**), alanine aminotransferase; AST (**B**), aspartate aminotransferase; ALP (**C**), alkaline phosphatase; BUN (**D**), blood urea nitrogen; CREA (**E**), creatinine; WBC (**F**), white blood cells; RBC (**G**), red blood cells; HGB (**H**), hemoglobin; HCT (**I**), hematocrit; MCV (**J**), mean corpuscular volume; MCH (**K**), mean corpuscular hemoglobin; MCHC (**L**), mean corpuscular hemoglobin concentration. Data are presented as the mean ± SD ($n = 3$, $p > 0.05$).

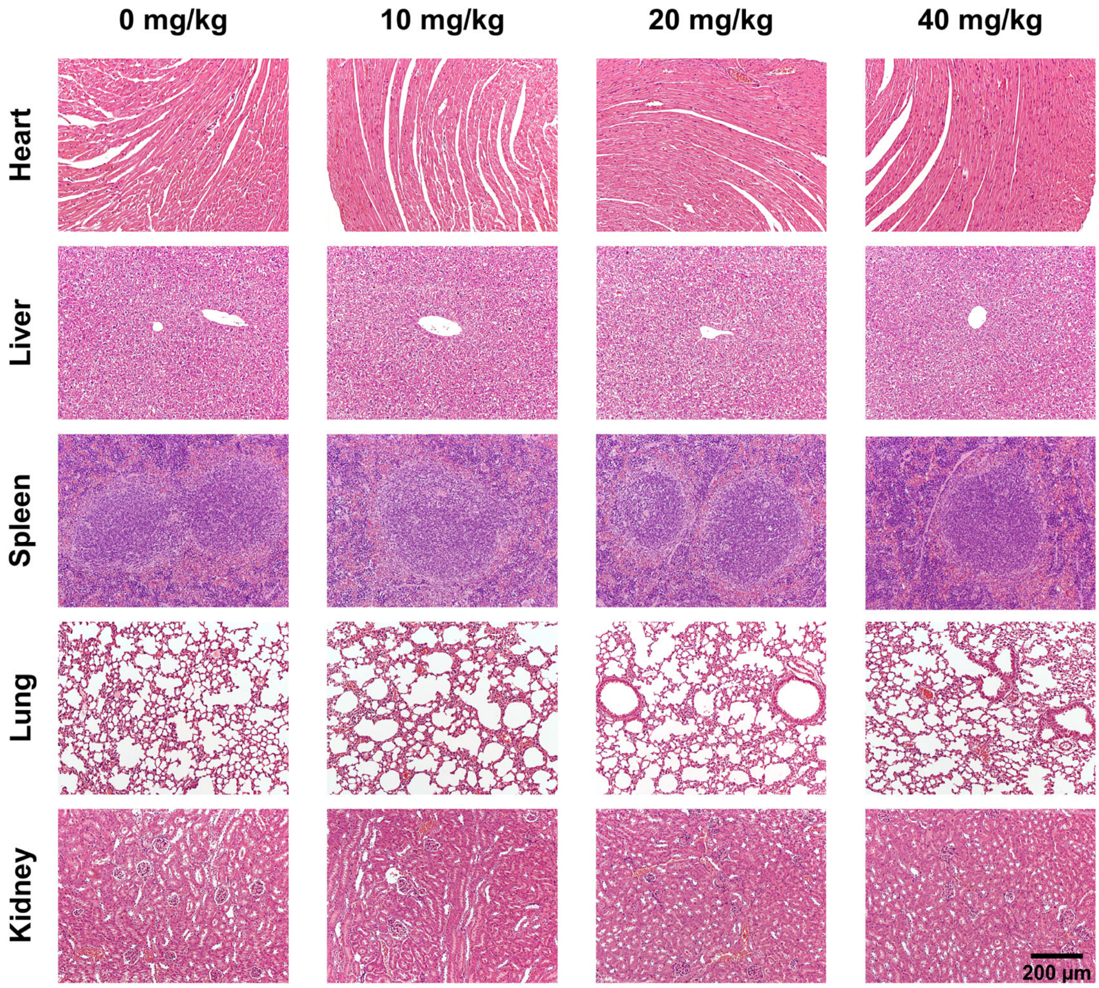

Figure S14. Hematoxylin and eosin (H&E) stained sections of major organs (the heart, liver, spleen, lung and kidney) from BALB/c mice before intravenous administration of CaO_2@HMSNs-PAA (n = 3). Scale bar, 200 μm.

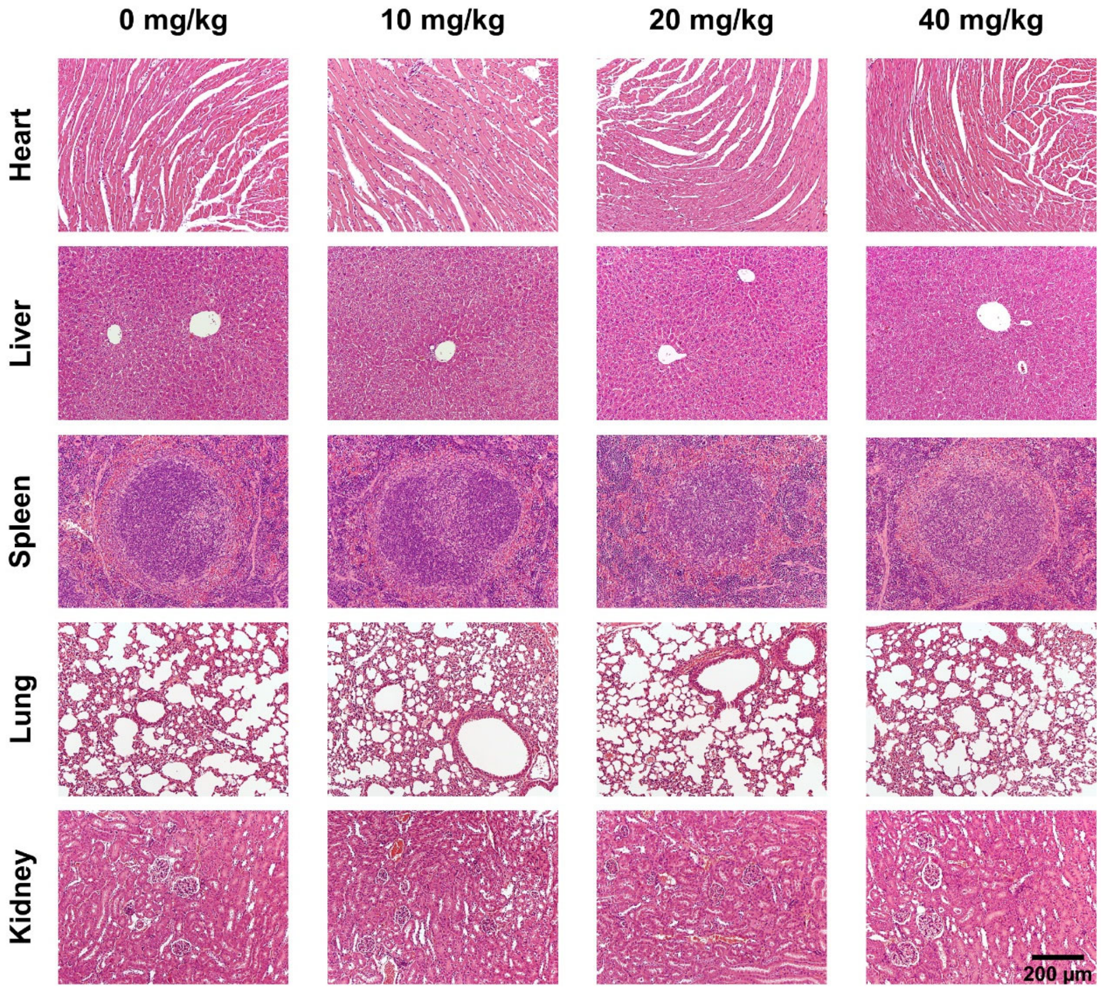

Figure S15. Hematoxylin and eosin (H&E) stained sections of major organs (the heart, liver, spleen, lung and kidney) from BALB/c mice on day 1 after intravenous administration of CaO_2@HMSNs-PAA with doses of 0 mg/kg, 10 mg/kg, 20 mg/kg and 40 m/kg (n = 3). Scale bar, 200 μm.

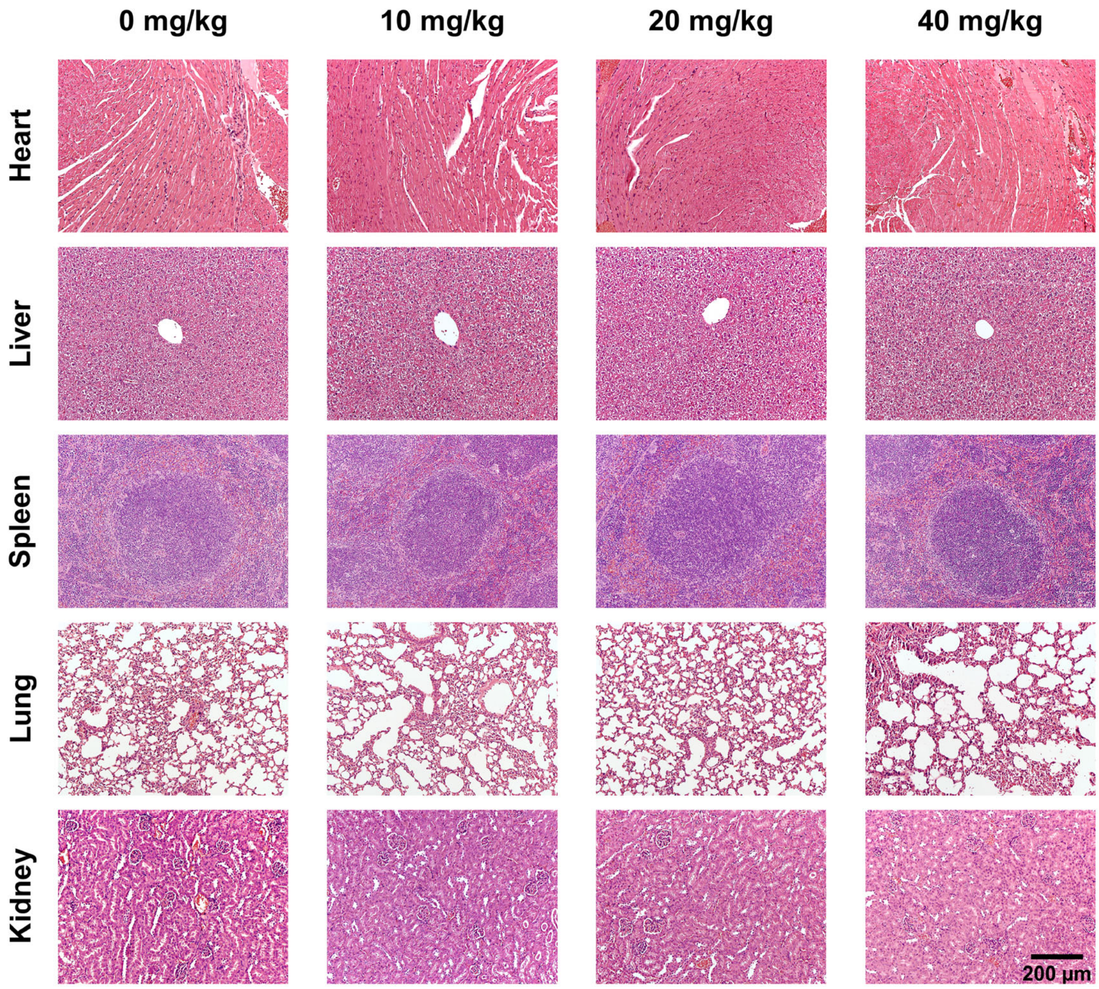

Figure S16. Hematoxylin and eosin (H&E) stained sections of major organs (the heart, liver, spleen, lung and kidney) from BALB/c mice on day 7 after intravenous administration of CaO_2@HMSNs-PAA with doses of 0 mg/kg, 10 mg/kg, 20 mg/kg and 40 m/kg (n = 3). Scale bar, 200 µm.

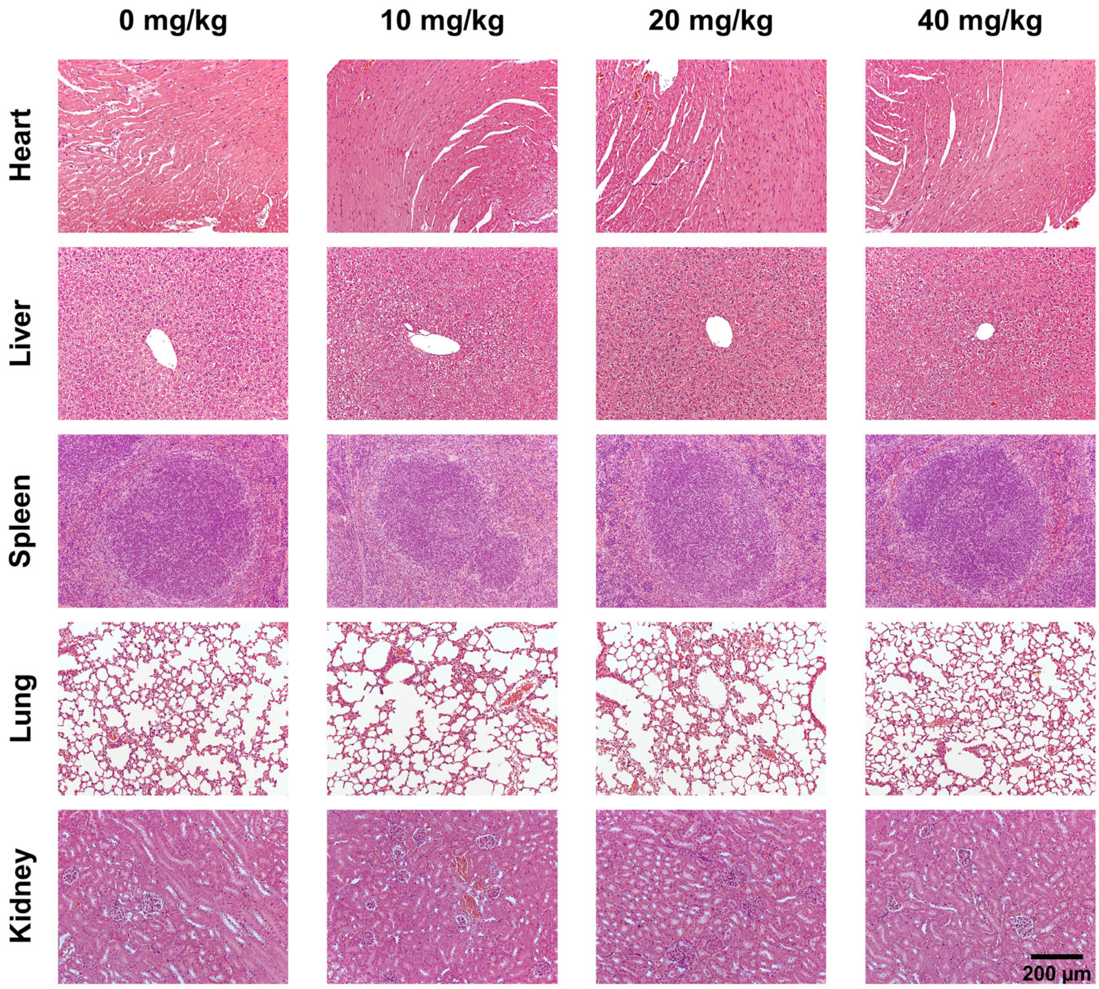

Figure S17. Hematoxylin and eosin (H&E) stained sections of major organs (the heart, liver, spleen, lung and kidney) from BALB/c mice on day 30 after intravenous administration of CaO_2@HMSNs-PAA with doses of 0 mg/kg, 10 mg/kg, 20 mg/kg and 40 m/kg (n = 3). Scale bar, 200 μm.

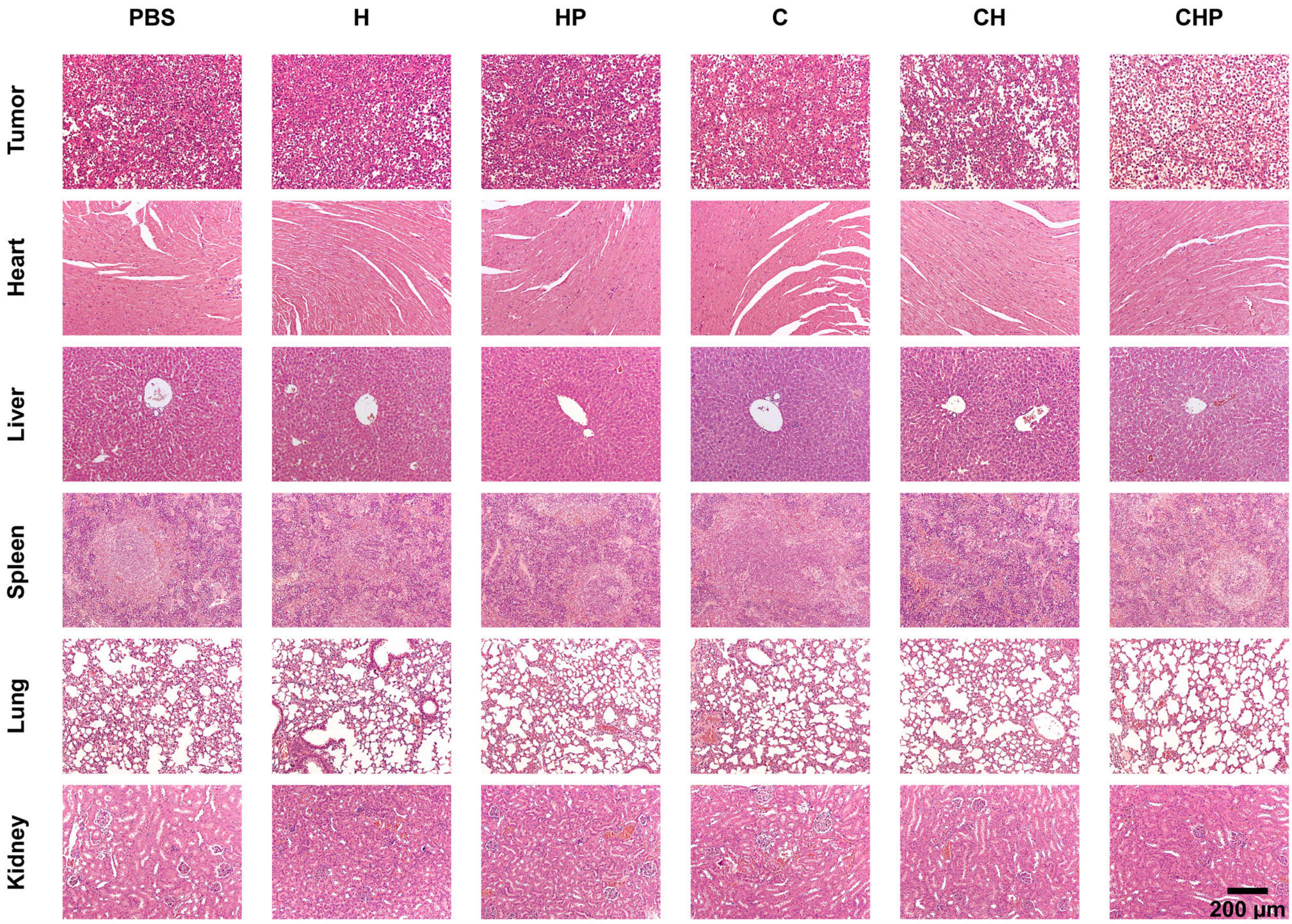

Figure S18. Histological changes of the PC-3 xenografted tumors and major organs after intravenous administration of CaO_2@HMSNs-PAA. Hematoxylin and eosin (H&E) stained sections of the tumors and major organs (the heart, liver, spleen, lung and kidney) from subcutaneous PC-3 xenografted tumor-bearing BALB/c nude mice on day 14 after intravenous administration of phosphate-buffered saline (PBS), HMSNs (H), HMSNs-PAA (HP), CaO_2 (C), CaO_2@HMSNs (CH) and CaO_2@HMSNs-PAA (CHP) (n = 5). Scale bar, 200 μm.

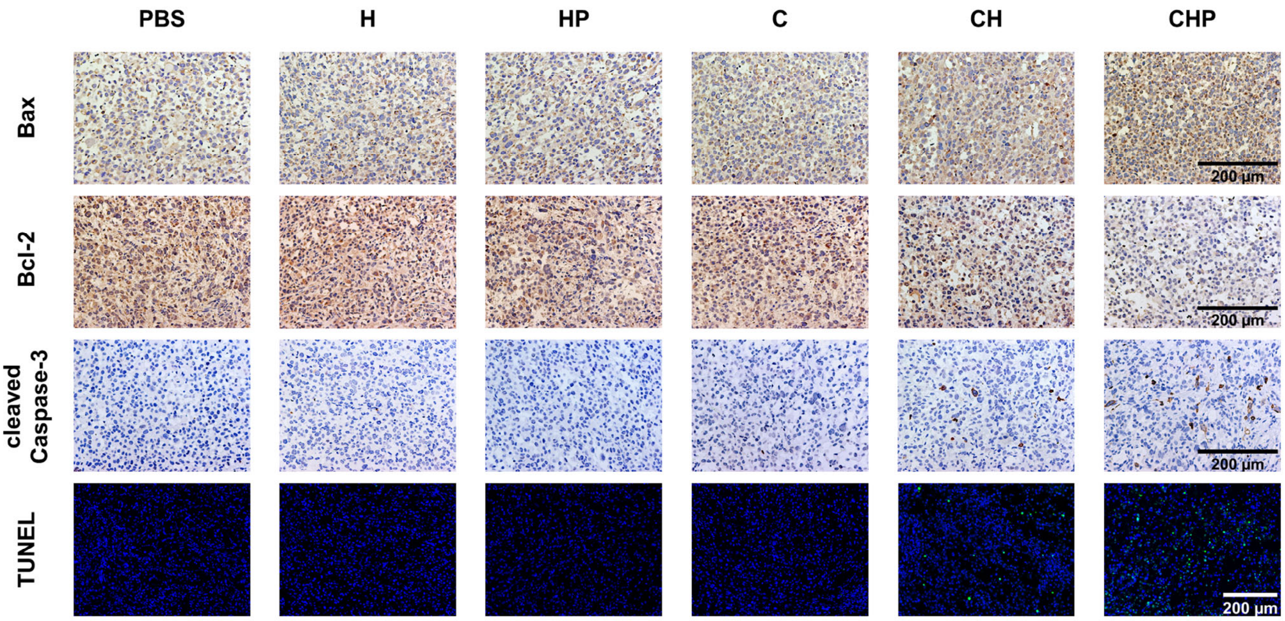

Figure S19. Immunohistochemical and TUNEL analysis of the PC-3 xenografted tumors after intravenous administration of CaO_2@HMSNs-PAA. Immunohistochemically stained images of the antigens (Bax, Bcl-2 and cleaved Caspase-3) and TUNEL analysis in the tumors from subcutaneous PC-3 xenografted tumor-bearing BALB/c nude mice on day 2 after intravenous administration of phosphate-buffered saline (PBS), HMSNs (H), HMSNs-PAA (HP), CaO_2 (C), CaO_2@HMSNs (CH) and CaO_2@HMSNs-PAA (CHP) (n = 3). Scale bars, 200 µm.
